# Bispecific antibodies and nanotechnology: a strategic alliance in cancer immunotherapy

**DOI:** 10.1186/s12943-025-02501-9

**Published:** 2025-11-17

**Authors:** Elisa Battistini, Philipp Lapuhs, Alberto Jiménez, Sergio Garrido-Areal, Lucía Rivas-Gómez, Ivana Zagorac, Luis Álvarez-Vallina, María José Alonso, Lucía Sanjurjo

**Affiliations:** 1 Department of Pharmacology, Pharmacy, and Pharmaceutical Technology, School of Pharmacy/Center for Research in Molecular Medicine and Chronic Diseases (CiMUS), University of Santiago de Compostela (USC), Santiago de Compostela, Spain; 2 Advanced Therapies Manufacturing Center, Galaria EPSS SA, Santiago de Compostela, Spain; 3 Cancer Immunotherapy Unit (UNICA), Immuno-oncology and Immunotherapy Group, Biomedical Research Institute H12O/H12O-CNIO Cancer Immunotherapy Clinical Research Unit/CNIO-HMRIB Cancer Immunotherapy Clinical Research Unit, Hospital Universitario 12 de Octubre (H12O), Spanish National Cancer Research Centre (CNIO), Hospital del Mar Research Institute Barcelona (HMRIB), Madrid & Barcelona, Spain; 4 Banc de Sang i Teixits, Barcelona, Spain; 5 Madrid Institute for Advanced Studies in Nanoscience, IMDEA Nanociencia, Madrid, Spain; 6 Health Research Institute of Santiago de Compostela (IDIS), Santiago de Compostela, Spain

**Keywords:** Bispecific antibodies, Nanotechnology, Targeting, mRNA, Cancer immunotherapy.

## Abstract

Bispecific antibodies (bsAbs), designed to recognize two distinct antigens or epitopes, enable innovative mechanisms of action for emerging generations of cancer immunotherapies. Despite their potential, bsAb therapeutics face several challenges related to their biodistribution and pharmacokinetics, which often result in a suboptimal efficacy/toxicity balance. Starting with a brief description of the relevance of bsAbs in cancer immunotherapy, this review aims to critically analyze the synergistic potential of nanotechnology and bsAb technology oriented to enhance therapeutic efficiency while reducing toxicity. This synergy can be achieved through several strategies: (i) bsAbs may function as targeting ligands to improve the biodistribution of drug-loaded nanocarriers; (ii) therapeutic bsAbs incorporated into nanocarriers may easily overcome biological barriers and reach their target; and (iii) bsAbs can be generated in vivo using mRNA-loaded nanocarriers encoding them. This review addresses challenges in these emerging areas and provides insights into future directions for this promising field.

## Introduction

Bispecific antibody (bsAb)-based immunotherapeutics have gained significant attention in cancer immunotherapy for their versatility and ability to achieve therapeutic effects that may surpass those of conventional antibodies, considering their potential to overcome resistance and enhance target specificity. The strength of bsAbs lies in their capacity to simultaneously bind two distinct antigens or epitopes, enabling a multitargeting approach. This feature is especially valuable for addressing the complexity of diseases involving multiple receptors, ligands or signaling pathways. The growing number of approved bsAbs (Table 1), along with a robust pipeline of over 200 candidates in clinical development [1], underscores their great potential in cancer therapy and diagnosis.

**Table 1:** BsAbs approved by EMA and/or FDA or currently in fast-track designation for cancer immunotherapy

Fist approval	Trade Name	BsAb name	Targets	Type	Indication
2009 (withdrawn 2017)	Removab	Catumaxomab	EpCAM x CD3	TCE	Ovarian ascites
2014	Blincyto	Blinatumomab	CD19 x CD3	TCE	Philadelphia chromosome-negative relapsed or refractory B cell precursor acute lymphoblastic leukaemia
2021	Rybrevant	Amivantamab	EGFR x cMet	Signaling inhibitor	Locally advanced or metastatic non-small cell lung cancer
2022	Kimmtrak	Tebentafusp	gp100-HLA-A*02 × CD3	TCE	Unresectable or metastatic uveal melanoma
2022	Tecvayli	Teclistamab	BCMA x CD3	TCE	Relapsed or refractory multiple myeloma
2022 (China)	Kaitanni	Cadonilimab	PD-L1 x CTLA-4	Checkpoint inhibitor	Hepatocellular carcinoma
2022	Lunsumio	Mosunetuzumab	CD20 x CD3	TCE	Relapsed or refractory follicular lymphoma
2023	Epkinly	Epcoritamab	CD20 x CD3	TCE	Relapsed or refractory diffuse large B-cell lymphoma
2023	Columvi	Glofitamab	CD20 x CD3	TCE	Relapsed or refractory diffuse large B-cell lymphoma or large B-cell lymphoma
2023	Elrexfio	Elranatamab	BCMA x CD3	TCE	Relapsed or refractory multiple myeloma
2023	Talvey	Talquelamab	GPRC5D x CD3	TCE	Relapsed or refractory multiple myeloma
2024	Imdelltra	Tarlatamab	DLL3 x CD3	TCE	Small cell lung cancer
2024	Ziihera	Zanidatamab	HER2 x HER2	Signaling inhibitor	HER2-positive biliary tract cancer
2024	Bizengri	Zenocutuzumab	HER2 x HER3	Signaling inhibitor	Non-small cell lung cancer and pancreatic adenocarcinoma
2024 (EU)	Ordspono	Odronextamab	CD20 x CD3	TCE	Relapsed/refractory follicular lymphoma or diffuse large B-cell lymphoma
2024 FDA Fast-track designation	-	Linvosentamab	BCMA x CD3	TCE	Relapsed or refractory multiple myeloma
2024 FDA Fast-track designation	-	Ivonescimab	PD-1 x VEGF	Signaling/ Checkpoint inhibitor	Non-small cell lung cancer
2024 FDA Fast-track designation	-	PT217	DDL3 x CD47	Signaling inhibitor	Small cell lung cancer and neuroendocrine prostate cancer
2024 FDA Fast-track designation	-	CTX-009	DLL4 x VEGFA	Signaling inhibitor	Metastatic or locally advanced biliary tract cancer
2024 FDA Fast-track designation	-	IBI363	PD-1 x IL2	Signaling/ Checkpoint inhibitor	Unresectable locally advanced or metastatic melanoma

*BCMA* B-cell maturation antigen, *CD* cluster of differentiation, *cMET* tyrosine kinase mesenchymal–epithelial transition, *CTLA-4* cytotoxic T-lymphocyte–associated antigen 4, *DLL* delta-like ligand, *EGFR* epidermal growth factor receptor, *epcam* epithelial cell adhesion molecule, *gp100* glycoprotein 100, *GPRC5D* G protein–coupled receptor class C group 5 member D, *HER* human epidermal growth factor receptor, *HLA-A* human leukocyte antigen A, *IL2* Interleukin 2, *PD-1* programmed death 1, *PD-L1* programmed death ligand 1, *TCE* T cell engager, *VEGFA* vascular endothelial growth factor A

**Fig. 1:**
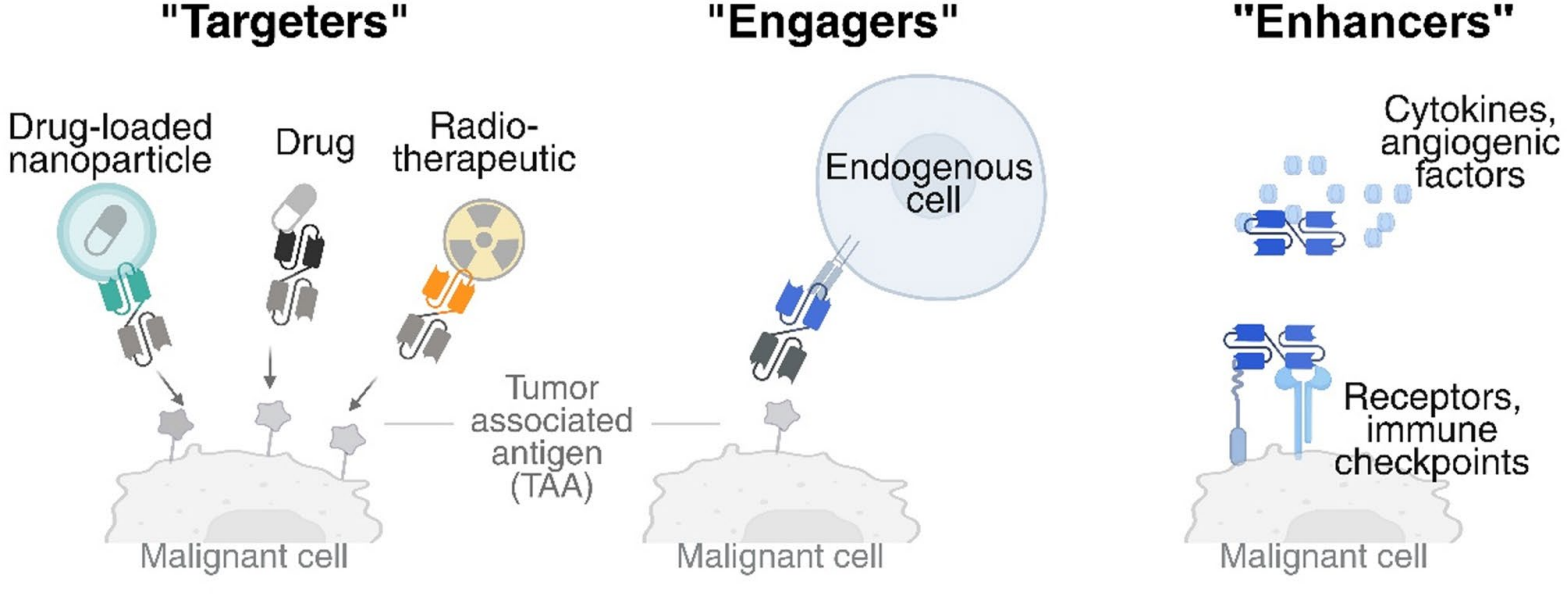
Mechanisms of action of bsAbs. Functional classification of bispecific antibodies (bsAbs) in cancer therapy. “Targeters”: direct therapeutic agents (e.g., drugs, nanoparticles, radiotherapeutics) to tumor-associated antigens (TAA). “Engagers”: recruit immune effector cells to malignant cells. “Enhancers”: modulate the tumor microenvironment by targeting cytokines, angiogenic factors, receptors, or immune checkpoints. Created in https://BioRender.com

BsAbs are available in a variety of formats [2] and can mediate anticancer effects through several molecular mechanisms of action (MoA). These MoA, reviewed below, include delivering therapeutic payloads to a specific target (“targeters”), bridging two cell types (“engagers”), or engaging two molecules on the same cell membrane or in the tumor microenvironment (“enhancers”) (Fig. 1) [1, 3].

- “Targeters”

Targeted delivery of payloads, including chemotherapeutics or radiotherapeutics, and nanoparticles (NPs) for cancer treatment or diagnosis can be facilitated by the bsAb technology [4]. Their multi-binding capability can be leveraged in both targeting or pre-targeting strategies, where one arm can bind the cargo while the other directs it to a tumor-associated antigen (TAA), or both arms can engage distinct TAAs to enhance tumor specificity. In addition, bsAbs can enhance payload internalization and intracellular trafficking by targeting transmembrane proteins, such as the prolactin receptor (PRLR), CD63, or cytokine receptors, thereby increasing uptake into tumor cells [5]. These approaches are being investigated to improve the efficacy of radiotheranostics, bsAb-drug conjugates, and nanomedicines, particularly in heterogeneous tumor populations.

- “Engagers”

Multiple bsAbs have been developed to bring two cell types together, primarily to transiently connect cancer cells with cytotoxic T cells. Bispecific T cell engagers (TCEs) accomplish this dual targeting by simultaneously binding to a selected TAA on the cancer cell surface and to the extracellular CD3 subunit on the T cell surface, thereby inducing specific T cell-mediated tumor cell killing. As shown in Table 1, most approved TCEs are indicated for hematological malignancies and have shown their ability to target CD19 in B-cell precursor acute lymphoblastic leukemia (B-ALL), CD20 in non-Hodgkin lymphoma, B-cell maturation antigen (BCMA), and G protein–coupled receptor GPRC5D in multiple myeloma. A key factor in their success is that, although these targets are present on regular blood cells, their depletion can be tolerated without causing severe adverse effects. Progress with TCEs in the treatment of solid tumors has been slower, likely due to the heterogeneity of tumor antigens and the immunosuppressive nature of the tumor microenvironment (TME) [6]. In this context, the TCE Tebentafusp has shown a significant improvement in overall survival in patients with solid tumors. This effect is mediated by the recognition of a peptide fragment of gp100, an intracellular melanoma-associated antigen, presented by HLA-A2:01 molecules on the surface of cancer cells [7]. While T cells have dominated the field of cell-engagers, significant efforts are underway to develop next-generation bsAbs engaging other immune cells such as natural killer (NK) cells, dendritic cells, neutrophils, or macrophages/monocytes [8].

- “Enhancers”

Another application of bsAbs in cancer therapy is related to the restoration and enhancement of antitumor immunity. Among these approaches, bsAbs can block two interrelated signaling pathways by targeting two epitopes on tumor cells or in the TME. The antigen pairs may include angiogenic factors, tyrosine kinase receptors, cytokines, or cytokine receptors [9]. An example of the success of this approach is illustrated by Amivantamab (Rybrevant), which targets epidermal growth factor (EGFR) and tyrosine kinase receptors (c-MET) (EGFR x c-MET) and was approved in May 2021 for non-small cell lung cancer treatment [10]. Another common design principle is the simultaneous targeting of two immune checkpoints, thereby reducing the probability of resistance, and potentially achieving better efficacy compared to monotherapy in solid tumor treatment [11]. Cadonilimab, a programmed cell death ligand 1 (PD-L1) and cytotoxic T lymphocyte associated protein 4 (CTLA-4) (PD-L1 x CTLA-4) blocker, became the first dual immune checkpoint inhibitor bsAb. It was approved in China in 2022 for the treatment of relapsed or metastatic cervical cancer [12]. Most of the recent fast-track designations are for this class of bsAbs, illustrating their active investigation and progress toward clinical application. Novel combinations are also arising, including the anti-PD-1/interleukin-2 (IL-2) bsAb fusion protein, IBI363, that achieves simultaneous PD-1 blockade and IL-2 delivery for melanoma treatment.

**Fig. 2:**
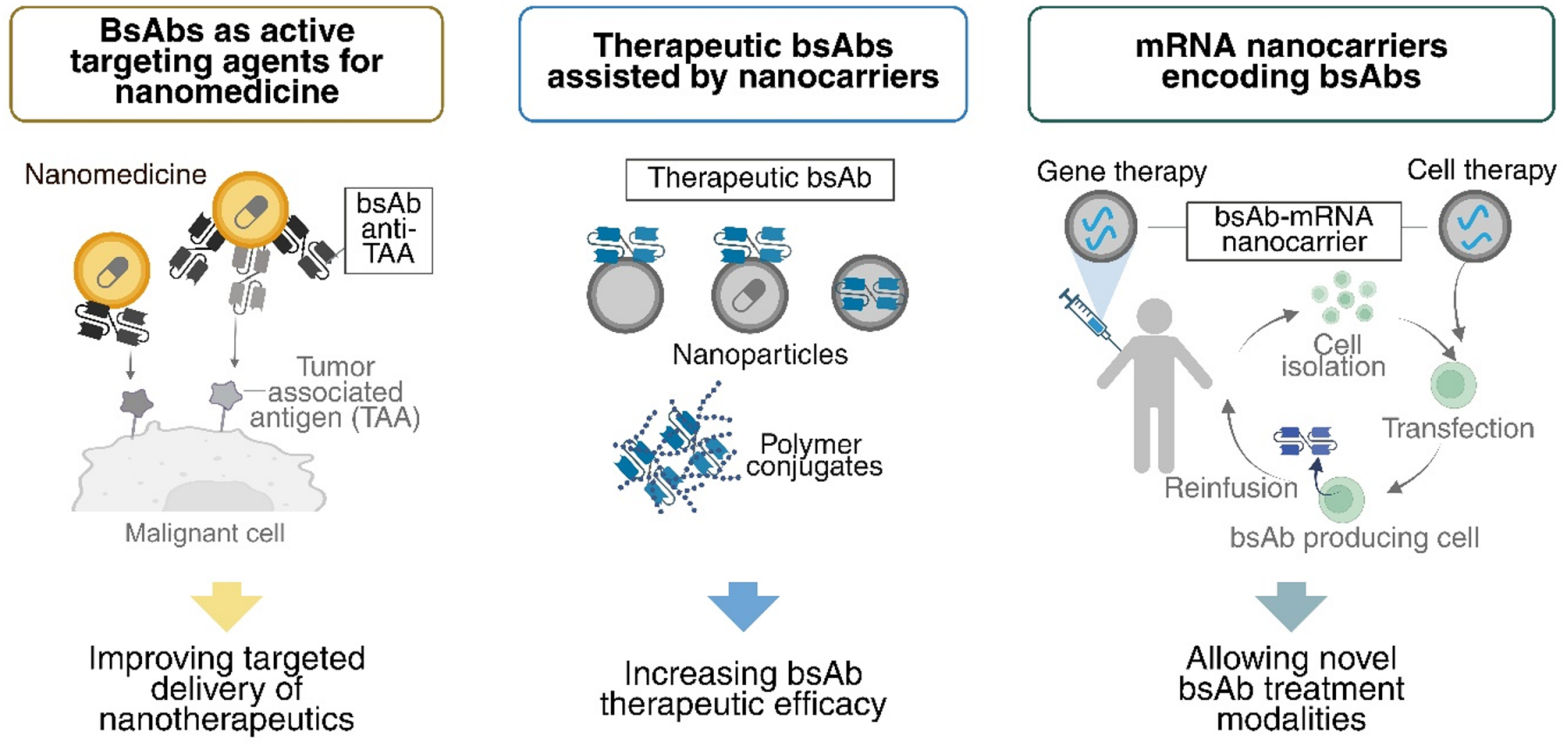
Synergies between bsAbs and nanotechnology in cancer therapy. Nanomedicine strategies leveraging bsAbs include: bsAbs as active targeting ligands to direct nanocarriers to TAAs (left), therapeutic bsAbs incorporated into or assembled with nanocarriers aimed to increase bsAb therapeutic efficacy (middle), mRNA-loaded nanocarriers encoding bsAbs enabling gene or cell therapy approaches (right). Created in https://BioRender.com

This review explores how advances in nanotechnology and bsAb technology can synergistically benefit cancer therapy (Fig. 2). First, we examine the potential of bsAbs as active targeting agents in nanomedicine to improve tumor specificity. We then discuss how nanotechnology can overcome key limitations of bsAb therapies, including limited half-life and instability, ultimately advancing their clinical translation.

## BsAbs as targeting agents for drug-loaded nanocarriers

A major limitation to the antitumor efficacy of drug-loaded NPs is their off-target accumulation in organs such as the liver and spleen [13, 14]. While monoclonal antibodies (mAbs) have been widely employed as targeting ligands, antibody-conjugated NPs have yet to reach clinical translation [15]. In this context, the multi-specificity of bsAbs may offer additional advantages. By simultaneously binding two distinct TAAs, bsAbs can increase tumor specificity and NP retention. Moreover, incorporating a bsAb domain that recognizes a NP moiety can streamline NP functionalization [16].

Various types of NPs functionalized with bsAbs have been investigated, including liposomes, lipid nanoparticles (LNPs), and polymeric NPs. These formulations are primarily designed for the targeted delivery and protection of therapeutic nucleic acids, or cytotoxic agents like doxorubicin and docetaxel, linked to significant side effects. An overview of these strategies is presented in Table 2. Approaches for active targeting using bsAbs are discussed in two main categories: bsAb conjugation to the NP surface and pre-targeting delivery.

**Table 2:** BsAb-based active targeting strategies in nanomedicine

Nanocarrier	Payload	Ab format	Targets	NP functionalization	Tumor model	Ref
Liposome	-	bispecific sdAb	HER2 (bivalent)	Covalent (maleimide-thiol)	HER-positive cancer cell lines	[17]
Manganese-doped iron oxide NP	Fluorescent dye (CyTE777)	bsAb	HER2 x EGFR	Covalent (EDC/NHS)	Breast and colorectal cancer	[18]
PEGylated liposome	Doxorubicin	bsAb	HER2 x PEG EGFR x PEG	Non-covalent	Colorectal cancer	[19]
PEGylated liposome	Doxorubicin	bsAb	HER2 x PEG	Non-covalent	Ovarian cancer	[20]
PEGylated liposome	Doxorubicin	bsAb	HER2 x PEG	Non-covalent	Breast cancer	[21]
PEGylated liposome	Doxorubicin	bsAb	CD20 x PEG CD22 x PEG CD38 x PEG	Non-covalent	Leukemia	[22]
PEG NP	-	bsAb	EGFR x PEG	Non-covalent	Breast cancer	[23, 24]
Polymeric NP	Doxorubicin	bsAb	HER2 x PEG	Non-covalent	Breast cancer brain metastasis	[25]
Polymeric NP	Doxorubicin	bsAb	Ephrin A2 x PEG	Non-covalent	Brain cancer	[26]
mPEGylated lecithin micelle	Docetaxel	bsAb	DNS x PEG HER2 x PEG	Non-covalent	Breast cancer	[27]
Micelle	Docetaxel	bsAb and TrAb	EGF3 x PEG FAP x PEG EGF3 x FAP x PEG	Non-covalent	Pancreatic cancer	[28]
PEGylated nano-emulsion	Docetaxel and Pictilisib	bsAb	HER x PEG HER-IV x PEG	Non-covalent	Breast cancer	[29]
Polymeric NP	Doxorubicin, camptothecin and imaging tracer (Cy5)	bsAb	EGFR x PEG	Non-covalent	Breast cancer	[30]
LNP	mRNA encoding PE38	bsAb	GRP78 x PEG	Non-covalent	Hepatocellular carcinoma	[31]
LNP	siRNA encoding PLK1	bsAb	EGFR x PEG	Non-covalent	High-risk neuroblastoma	[32]
LNP	Model mRNA	bsAb	PD-L1 x HA CD4 x HA CD5 x HA	Non-covalent	-	[33]
PEGylated liposome	Doxorubicin	bsAb	EGFR x PEG	Pre-targeting	Breast cancer	[34]
PEGylated liposome	Doxorubicin	bsAb	HER2 x PEG	Pre-targeting	Breast cancer	[35]
Polystyrene NP	Doxorubicin	bsAb	HER2 x PEG	Pre-targeting	Breast cancer	[36]
PEGylated liposome	Doxorubicin	bsAb	CD20 x PEG	Pre-targeting	Leukemia	[37]
LNP	Model mRNA	bsAb	EGFR x PEG	Non-covalent and pre-targeting	Breast cancer	[38]

*bsab* bispecific antibody, *bsabs* bispecific antibodies, *CD* cluster of differentiation, *DNS* Dansyl (negative control), *EDC* 1-ethyl-3-(3-dimethylaminopropyl) carbodiimide, *EGF* epidermal growth factor, *FAP* fibroblast activation protein, *GRP78* glucose-regulated protein 78, *HER* human epidermal growth factor receptor, *LNP* lipid nanoparticle, *mRNA* messenger RNA, *NHS* N-Hydroxy succinimide, *PEG* polyethylene glycol, *PLK1* polo-like kinase 1, *NP* nanoparticle, *sirna* small interfering RNA, *sdab* single domain antibody, *trab* trispecific antibody

### Functionalization of NPs with BsAbs

**Fig. 3:**
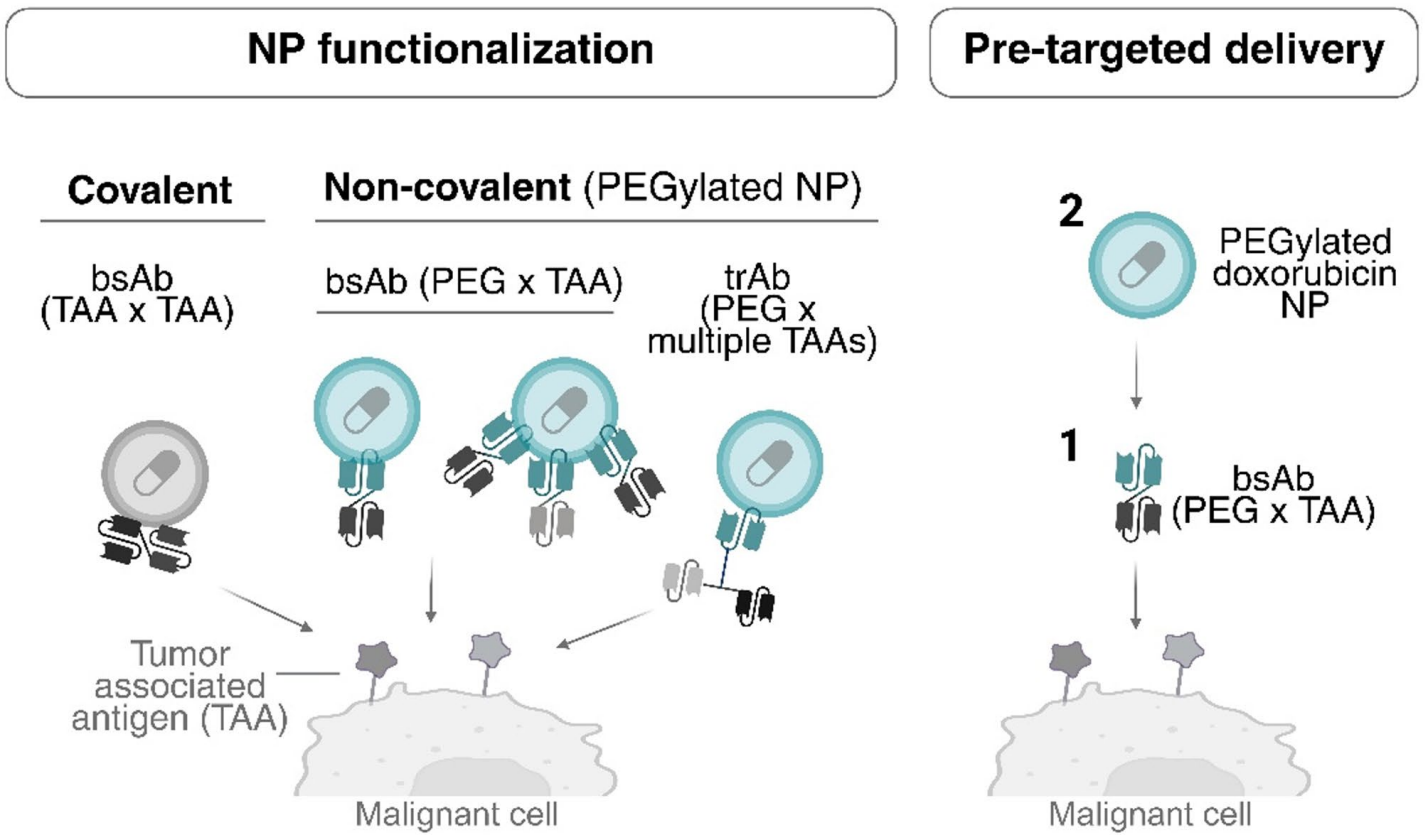
BsAbs as active targeting agents in nanomedicine. BsAbs used as targeting agents to functionalize drug-loaded nanocarriers either covalently, through non-covalent antigen-antibody interactions or allowing pre-targeted strategies. Created in https://BioRender.com

Among the different approaches for bsAb functionalization of NPs (Fig. 3) covalent functionalization has been scarcely explored. This is likely due to the need of antibody modification, as well as specific pH and media conditions, which may compromise both antibody and nanocarrier stability [39].

Despite these technical hurdles, liposomes functionalized with bivalent single-domain (sd) bispecific anti-HER antibodies have shown ~ 10-fold higher binding affinity to human epidermal growth factor receptor 2 (HER2) and up to 3-fold increased cellular uptake in HER-positive cancer cell lines compared to monospecific formats [17]. Covalent bsAb functionalization has also been applied in molecular imaging. For instance, iron oxide NPs functionalized with HER x EGFR bsAbs using carbodiimide chemistry enabled MRI-based tumor imaging in mouse models [18]. Although still limited, innovative approaches such as chemo-enzymatic methods are being explored for bispecific sdAb attachment to NPs [40].

Non-covalent strategies based on bsAbs with a domain specific towards NP surface moieties may facilitate NP functionalization. Typically, the anti-NP domain targets polyethylene glycol (PEG) on NP surfaces. Meanwhile, the second (or third, in case of trispecific Abs) binding domain is used to target cancer cells. This approach has been applied to several PEGylated nanocarriers, including liposomes, micelles, nanoemulsions, polymeric NPs, quantum dots and AuNPs [24–30, 41] and, also, to approved PEGylated nanomedicines such as liposomal doxorubicin (Doxil, Caelyx) [21, 22, 42, 43]. Preclinical studies showed that mixing anti-PEG x TAA bsAbs with Doxil enhanced tumor accumulation and therapeutic efficacy across various models, including ovarian, breast, colorectal cancers, and leukemia [19, 21, 22]. For example, intravenous injection of anti-PEG x HER2 bsAbs-targeted Doxil into mice bearing ovarian adenocarcinoma tumors, significantly increased the tumor fluorescence uptake signal by up to 240% at 72 h post treatment compared with isotype control bsAbs-Doxil. Moreover, HER2⁺ tumor growth was significantly inhibited compared with both isotype control bsAbs-Doxil and unmodified Doxil. Based on this conceptual evidence, there is increasing interest in implementing this strategy to improve the targeted delivery of therapeutic nucleic acids through non-covalently functionalized LNPs [31–33, 38].

Several studies have addressed key technical aspects of bsAb-functionalized NPs, providing insights into how bsAb surface density, PEG architecture, and multi-bsAb conjugation influence NP behaviour and offer strategies to maximize therapeutic outcomes.

The impact of anti-PEG bsAb density on the in vivo NP behavior was studied, for example, using PEGylated mesoporous silica NPs. BsAbs anti-mPEG x EGFR were attached via incubation and indirectly quantified by fluorescence, yielding 45 to 173 bsAbs per particle. Notably, radiolabelled particles with lower bsAb surface density showed a non-significant trend toward improved tumor accumulation, together with a significant reduction in spleen accumulation upon intravenous administration in mice bearing breast cancer tumors [24]. This may reflect that a high functionalization degree compromises stealth properties. Hence, accurate quantification of bsAbs per particle is crucial for elucidating correlations between bsAb density and tumor accumulation. However, the existing literature lacks rigorous characterization, as most quantification methods are indirect and parameters such as ligand density, orientation, and stability in physiological media are often not reported, leading to a poor correlation between bsAb number and tumor targeting or therapeutic outcome.

Several studies have highlighted the critical role of PEG density and architecture on NP functionalization via PEG recognition. Lecithin micelles prepared with DSPE-PEG2K or DSPE-PEG5K were compared for cellular uptake following bsAb (anti HER or DNS x PEG) binding. Here, longer PEG chains (5 K) improved uptake by ~ 25% compared to shorter chains (2 K), as measured by mean fluorescence intensity in flow cytometry using NPs loaded with a fluorescent dye, likely due to better display of the targeting agent [27]. Another study that made use of hyperbranched mPEG in PEG-based polymeric NPs, concluded that this structure facilitated the binding of anti-PEG bsAbs and, ultimately, in vivo efficacy in a breast cancer model. However, because no direct comparison between hyperbranched and linear mPEG was performed, no conclusions regarding their relative performance can be drawn [23]. The effect of different PEG-lipid anchors for bsAbs anti-PEG x anti-EGFR to Polo-Like Kinase 1 (PLK1) siRNA-LNPs was also investigated [32]. While all LNPs accumulated predominantly in the liver, tumor accumulation at 48 h post-administration showed a 4.3-fold increase for DSPE-PEG LNPs and a 1.9-fold increase for DMG-PEG LNPs, relative to the vehicle control. Despite lower tumor accumulation, DMG-PEG LNPs achieved greater therapeutic efficacy, reducing tumor size by 42.6% (*P* < 0.05) after 22 days in a neuroblastoma xenograft model. This might be explained by faster desorption of DMG-PEG, resulting in improved siRNA transfection. This illustrates the importance of balancing target accumulation with delivery efficiency. Collectively, these results underscore the importance of PEGylation parameters, such as chain length, branching and release kinetics, to fine-tune bsAb interaction and optimize therapeutic outcomes.

Another line of research has been the conjugation of multiple anti-TAA x PEG bsAbs to NPs. This strategy is especially promising in cases of loss of TAAs, a common resistance mechanism where tumor cells reduce or internalize specific surface markers and thereby evade recognition. Nanotechnology provides opportunities to mitigate this challenge by incorporating multiplexed targeting strategies into NP design by conjugating multiple anti-TAA×PEG bsAbs to the same platform, thereby broadening antigen recognition and maintaining therapeutic efficacy even in heterogeneous tumor environments. For example, Caelyx^®^ (a PEGylated liposome) was functionalized with single bsAbs (bsAb-Caelyx) or multiple bsAbs (Trio-Caelyx) to improve doxorubicin delivery in leukemia. In ALL-19 model of chemo-resistant B-cell acute lymphoblastic leukemia, Trio-Caelyx (functionalized with anti-CD19 x PEG, anti-CD22 x PEG, and anti-CD38 x PEG) improved event-free survival by 3.3-fold compared to PBS control. However, bsAb-Caelyx targeting CD22 alone achieved a 4.2-fold increase, outperforming Trio-Caelyx. Notably, a significantly greater accumulation of Trio-Caelyx was observed in the liver, suggesting that the addition of multiple bsAbs may enhance opsonization and clearance by the mononuclear phagocyte system [22]. However, in some studies it has been shown that the addition of multiple targeting ligands to NPs does not significantly enhance drug accumulation in the tumor [29], highlighting the need for comprehensive in vivo studies across different tumor models to assess their advantages over single anti-TAA strategies.

While PEGylated NPs have dominated the field of targeted cancer therapeutics, the mounting evidence of PEG-associated immunogenicity and reduced efficacy upon repeated administration has prompted investigation of alternative surface modification strategies [44, 45]. PEG alternatives, including zwitterionic polymers [46] or polysarcosines [47], or extracellular vesicle (EV)-derived membranes as NP coating material have been used to reduce PEG-associated immunogenicity [48, 49]. However, such alternatives are yet to be explored in the context of NP bsAb therapeutics. Recently, biotechnological engineering of dendritic cells enabled the generation of bispecific EVs co-expressing anti-CD19 scFv and PD1, thereby combining tumor antigen targeting with immune checkpoint blockade [50]. Such pre-functionalized EVs may represent a promising avenue in the development of bsAb-functionalized NPs.

### Pre-targeted delivery

Pre-targeted delivery strategies involve a two-step process. First, bsAbs are administered intravenously to bind TAAs on cancer cells; second, drug-loaded PEGylated nanocarriers are administered intravenously, designed to interact with anti-mPEG domains on pre-localized bsAbs [36]. This strategy has been successfully applied in clinical trials to enhance the delivery of radiolabelled peptides for cancer imaging and therapy [51]. This concept is also being applied in NP delivery, specifically to enhance the accumulation and payload release of PEGylated liposomal doxorubicin in different cancer cell types (Fig. 3, right) [52]. The efficacy of this approach has been shown in triple-negative breast cancer using anti-PEG x EGFR bsAbs [34], in HER2-positive breast adenocarcinoma with anti-HER2 x anti-mPEG bsAbs [35, 36], and in lymphoma with anti-CD20 x anti-mPEG bsAbs [37]. Therefore, this strategy holds the potential to broaden the application of FDA-approved PEGylated therapeutics to different tumor types without the need for drug re-engineering. This is highly promising; however, the timing and pharmacokinetic coordination of the two-step administration are critical and must be carefully optimized in clinical settings.

A recent study compared non-covalent functionalization and pre-targeting strategies using anti-PEG x HER bsAbs, analyzing the biodistribution of luciferase mRNA-LNPs in mice bearing EGFR^+^ human tumor xenografts [38]. The pre-targeting strategy, with temporal separation of bsAb and LNP administration, resulted in a comparable increase in mRNA expression in tumor tissue (7-fold) relative to covalent functionalization (8-fold), with pre-targeting maintaining sustained expression over 48 h, exhibiting only a 10% decrease compared to a 60% reduction observed with covalent functionalization. Moreover, pre-targeting achieved a greater reduction in off-target delivery. These differences were linked to altered physicochemical properties of LNPs, including a shift from positive to negative surface charge as well as abundant protein corona formation in pre-functionalized LNPs.

While conjugation provides a one-step approach, bsAb attachment modifies nanocarrier surface properties, alters the resulting protein corona, and complicates manufacturing by requiring highly consistent conjugation chemistry. In contrast, pre-targeting strategies can reduce off-target delivery; however, their two-step administration introduces clinical challenges, including greater procedural complexity. Covalent systems are generally more reproducible across batches, while pre-targeting approaches may display higher variability in pharmacokinetics. Thus, selecting between these strategies involves carefully balancing efficacy, safety, reproducibility, and clinical feasibility.

### Enhancing targeting with BsAbs in nanomedicine. Critical considerations and future directions

The functionalization of NPs with bsAbs has been reported exclusively at the preclinical level, where studies consistently demonstrate enhanced antitumor efficacy. This observation is primarily attributed to increased tumor accumulation and prolonged payload retention. However, their impact on overall NP biodistribution and systemic toxicity remains unclear. Biodistribution studies in tumor models following intravenous administration have generally failed to significantly modify the distribution pattern, although they have provided an increased accumulation in the tumor tissue [20, 27, 29, 36]. This suggests that, while bsAb functionalization improves tumor accumulation, off-target distribution and associated side-effects may persist. A commonly investigated systemic side effect of nanomedicines is chemotherapy-derived cardiotoxicity, a dose-limiting factor that significantly impacts both treatment response and quality of life [53]. Among the limited studies that have systematically evaluated this side effect, most have not observed a significant improvement upon functionalization with bsAbs [21, 26, 30]. This suggests that functionalization alone may be insufficient to mitigate the principal systemic side-effects of nanomedicines. In contrast, polymeric NPs loaded with doxorubicin and functionalized with bsAbs (anti-HER3 x PEG) showed significantly reduced heart accumulation and cardiotoxicity [25]. These findings suggest the need for future studies to comprehensively determine biodistribution and systemic toxicity to assess whether bsAb-functionalized NPs can truly overcome these challenges. In fact, some studies suggests that bsAb functionalization does not markedly alter systemic biodistribution, potentially due to rapid bsAb desorption, protein corona masking, or suboptimal ligand orientation and density. Such factors may limit effective target engagement during circulation, underscoring the importance of optimizing surface engineering to enable more selective and safer NP delivery.

## BsAbs as therapeutic agents incorporated in nanocarriers

The anticipated widespread use of bsAb therapeutics presents significant challenges for clinical implementation, particularly due to their short plasma half-life and associated adverse effects such as cytokine release syndrome (CRS), neurotoxicity, cytopenia, and infections [54]. In terms of bsAb design, multiple strategies are being pursued to mitigate bsAb-related toxicities, particularly in TCE therapies. A key approach is the fine-tuning of anti-CD3 affinity to reduce excessive T cell activation and thereby minimize CRS [55, 56]. Building on this, recent advances in tri- and tetra-specific antibody (tsAb, ttsAb) engineering seek to overcome different limitations by incorporating: (i) recognition of multiple tumor epitopes to enhance avidity and reduce antigen escape [57], (ii) agonistic or costimulatory domains (e.g., 4-1BBL, OX40L, CD28) to sustain T-cell function and prevent exhaustion, and (iii) cytokine-blocking modules (e.g., IL-6R or IL-1R antagonists) to dampen inflammatory cascades and reduce CRS risk [57, 58]. Another promising avenue is the development of conditionally active TCEs, which remain inactive until activated within the TME, either through protease cleavage, pH-sensitivity, or controlled-release prodrug designs. Such approaches are intended to restrict T cell engagement to tumor sites, thereby improving safety and preventing off-tumor toxicities [59–62]. Collectively, these innovations highlight how rational bsAb engineering is reshaping the therapeutic index of next-generation TCEs.

The short plasma half-life of small bsAbs often requires continuous infusion to maintain therapeutic levels. For example, Blinatumomab is administered via continuous intravenous infusion through an implantable pump for 4 weeks [63]. Strategies to prolong bsAb half-life include genetic fusion or chemical conjugation to long-circulating proteins like IgG or albumin [64], as well as the synthesis of bsAb-polymer conjugates with materials like PEG or poly-ADP-ribose [65, 66]. Local administration may also improve efficacy in certain clinical contexts. A feasible approach involves solid implants or injectable depots made from biocompatible polymers. For example, subcutaneously injectable PEGylated poly (lactic acid) (PEG-PLA) [67] or PEGylated poly (lactic acid-co-glycolic acid) (PEG-PLGA) [68] polymeric depots incorporating bsAbs have shown sustained release in plasma and improved antitumoral efficacy in prostate and breast cancer xenograft models, respectively.

Nanocarriers present an alternative strategy to improve bsAb pharmacokinetics and biodistribution, potentially reducing the required dose and minimizing adverse effects. Beyond delivery optimization, NPs can facilitate combinational therapies by carrying multiple agents within a single platform. This versatility provides opportunities to overcome resistance mechanisms, such as antigen loss and tumor-induced immunosuppression. Through combinatorial or sequential targeting, nanocarriers may help counteract antigen escape, while the localized co-delivery of bsAbs with immunomodulators could remodel the immunosuppressive tumor microenvironment and enhance therapeutic efficacy.

### Technical approaches for loading BsAbs into nanocarriers

**Fig. 4:**
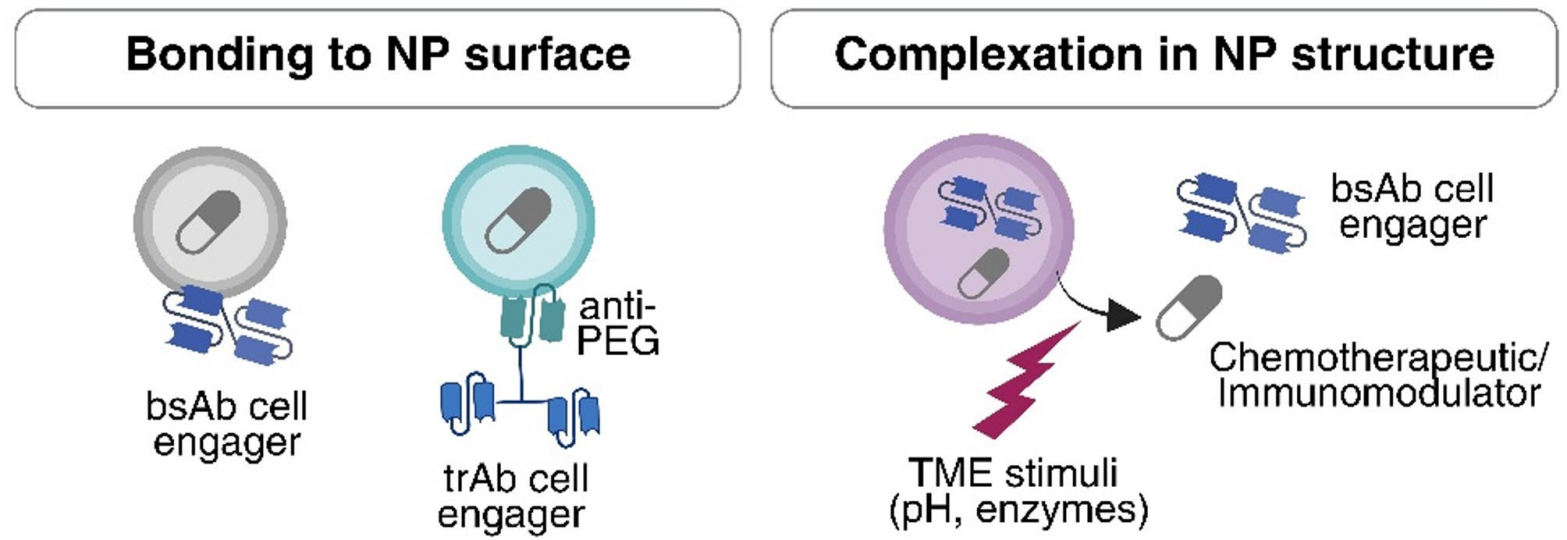
BsAbs as therapeutic agents in nanomedicine. BsAbs incorporated into nanocarriers, either by surface attachment or by complexation within the nanoparticle structure, allowing controlled release in response to tumor microenvironment (TME) stimuli. Created in https://BioRender.com

Most research to date has focused on decorating nanocarriers with multiple classical mAbs to generate bispecific platforms, which have demonstrated favorable pharmacokinetics, and improved antitumor efficacy [69]. In contrast, the incorporation of bsAbs or trispecific antibodies (trAbs) into NPs remains comparatively underexplored. Several strategies have been used to attach or complex bsAbs to inorganic, hybrid, lipidic, and tumor-responsive nanocarriers (Fig. 4). In these studies, therapeutic cell engager bsAbs are incorporated into NPs that simultaneously support combination therapies enhancing their overall antitumor activity.

For example, Xu et al. covalently conjugated a novel NK cell-engager (NKCE) bsAb targeting the NK cell marker CD16 and the tumor biomarker carcinoembryonic antigen (CEA) to the surface of PEGylated hollow mesoporous ruthenium NPs. These constructs, intended to function as photothermal therapy agents, were additionally loaded with the fluorescent anti-tumor complex RBT. In vivo studies in a colorectal tumor model showed significantly superior tumor targeting and anticancer efficacy compared to RBT alone, free bsAb, or the combination of RBT-loaded NPs and free bsAb, achieving up to 96% tumor growth inhibition after three injections combined with near-infrared irradiation [70]. The enhanced therapeutic effect was attributed to the simultaneous delivery of RBT to solid tumors and bsAb-mediated immunotherapy. In contrast, another approach using NPs decorated with trAbs targeting CD3, PD-L1, and PEG did not show added benefits compared to free trAb or drug-loaded NPs alone in a breast cancer model [71]. The lack of synergy may result from suboptimal trAb exposure, impaired release, or functional inhibition of therapeutic components upon NP integration. These challenges highlight the importance of refining the design and functionalization of NPs to ensure therapeutic benefit.

Another promising approach incorporates bsAbs into tumor-responsive NPs that enable controlled release in response to TME properties, such as pH, hypoxia, or enzyme activity, including metalloproteinases (MMPs) [72]. Responsive NPs contained bsAbs targeting B7-H3 and CD3 were developed for glioblastoma (GBM) therapy. Their formulation included a multifunctional polymer composed of: (i) hyaluronic acid (HA), which targets CD44 receptors overexpressed in GBM; (ii) PLGLAG (proline-leucine-glycine-leucine-alanine-glycine), the tumor-responsive linker cleavable by MMP-2; and (iii) a dimer of epigallocatechin-3-O-gallate (EGCG), a polyphenol with attributed anticancer properties. Upon intravenous injection in a GBM mouse model, the bsAb-loaded NPs demonstrated higher tumor accumulation, greater inhibition of GBM growth and extended survival. Specifically, at day 56 post-treatment, survival rates showed a statistically significant improvement over controls. The therapeutic benefit observed in vivo was associated with reduction of proteins related to ferroptosis, B7-H3 downregulation, and increased intracranial infiltration of lymphocytes [73]. This illustrates the potential benefits of bsAb incorporation into rationally designed NPs.

A different strategy explored the incorporation of the approved bsAb blinatumomab into acid-labile polydopamine (PDA)-CaCO₃ NPs via electrostatic adsorption. These NPs were co-loaded with imiquimod (IMQ), a toll-like receptor 7 (TLR7) agonist. The payload was released in a controlled manner upon disintegration of the nanosystem in the acidic TME, while PDA conferred photothermal therapy capabilities. In a breast tumor model, the bsAb-loaded NPs significantly suppressed tumor growth without signs of systemic toxicity. Mechanistically, treatment with these NPs inhibited regulatory T-cell activity, promoted the infiltration of CD4⁺/CD8⁺ and IFN-γ⁺ cytotoxic T lymphocytes, enhanced IFN-γ secretion, and induced stronger T-cell activation, ultimately promoting a durable antitumor immune response [74].

### Nanotechnology for improving current BsAb therapeutics. Critical considerations and future directions

Although still at an early stage of development, nanotechnology-based strategies using bsAbs have shown improved efficacy over free bsAbs, particularly by enabling combinatorial treatments that engage multiple antitumor mechanisms simultaneously. These include combining bsAbs with chemotherapeutics or photothermal agents to promote immunogenic cell death, thereby enhancing antigen release and immune activation. In parallel, bsAb- nanocarrier platforms have been engineered to incorporate immune modulators such as TLR agonists or checkpoint inhibitors, which synergize to potentiate T cell responses and overcome the immunosuppressive TME [70, 71, 74]. Moreover, tumor-responsive nanoplatforms capable of releasing payloads in response to microenvironmental cues (e.g., pH shifts, protease activity) enable dynamic adaptation to tumor conditions. Such adaptive designs enhance efficacy while simultaneously reducing systemic toxicity, positioning nanotechnology as a powerful tool to overcome antigen escape and microenvironmental barriers in solid tumors. While the complexity of these systems may complicate the assessment of individual component contribution, their synergistic potential holds promise for enhancing antitumor responses and overcoming resistance.

The hypothesis that NP encapsulation may prolong the circulation time of bsAbs is conceptually compelling; however, currently, there is not clear evidence supporting this advantage. Regarding safety, only a few studies have assessed the toxicity of therapeutic bsAb-loaded nanoplatforms, generally reporting no signs of organ damage or abnormal body weight [70, 71, 73]. Nevertheless, it remains unclear whether NP-mediated delivery could mitigate severe adverse effects commonly observed in bsAb clinical trials, such as the CRS and neurotoxicity. Future investigations should emphasize rational NP engineering and direct comparison with free bsAbs to determine whether these platforms can effectively extend bsAb circulation half-life and reduce off-target effects.

BsAb development has primarily focused on well-established cell surface antigens such as CD19, HER2 or EGFR, as well as components of the TME including fibroblast activation protein (FAP). While these approaches have demonstrated significant clinical promise, they are often associated by the risk of “on-target, off-tumor” toxicity, resulting from low-level antigen expression on normal tissues. This has driven researchers to explore more specific targets. The majority of tumor-specific antigens are localized intracellularly and, therefore, remain largely inaccessible to conventional antibody therapies. Recent developments in bsAb-based therapies have focused on targeting intracellular antigens or neoantigens presented as peptide/MHC-I complexes on the surface of tumor cells [75]. A key innovation in this area has been the development of immune-mobilizing monoclonal TCRs against cancer (ImmTACs), which combine an engineered, high-affinity TCR specific for peptide–MHC complexes with an anti-CD3 scFv to redirect T-cell activity toward tumor cells. Tebentafusp, the first ImmTAC approved for metastatic uveal melanoma, has validated this concept in the clinic by demonstrating durable survival benefit despite low target antigen density [76]. In parallel, TCR-mimetic bispecific antibodies (TCR-mimic bsAbs) have been generated to bind intracellularly derived peptide–MHC complexes with antibody-like specificity, extending the reach of bsAb therapies to non-surface tumor antigens such as mutated p53 or KRAS [77, 78]. These strategies significantly expand the druggable tumor antigen repertoire, enabling the selective targeting of neoantigens and otherwise “undruggable” intracellular proteins. Collectively, ImmTACs and TCR-mimic bsAbs represent a new frontier in bsAb engineering, offering a route to precise, tumor-restricted therapies with transformative clinical potential. In parallel, nanotechnology is emerging as a key tool for the direct intracellular delivery of mAbs, overcoming traditional delivery barriers. This approach has shown success with mAbs targeting intracellular oncoproteins like mutant KRAS [79]. Building on these advances, bsAbs could be designed to simultaneously target multiple intracellular oncoproteins or signaling pathways, with nanocarriers facilitating their intracellular delivery in either protein or nucleic acid form. The RNA-based approach adds a new dimension to bsAb therapy and will be the focus of the following section.

## mRNA nanocarriers encoding therapeutic BsAbs

**Fig. 5:**
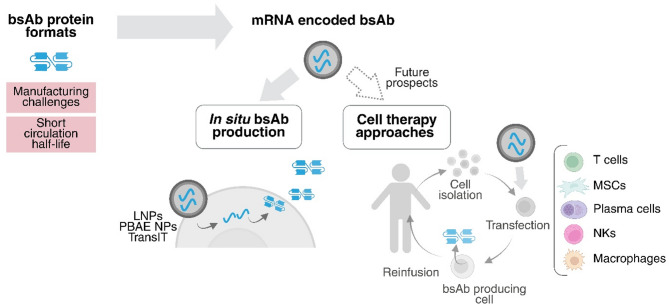
mRNA-encoded bsAb: In situ expression and emerging applications in cell therapy. Comparison between conventional bsAb protein formats and mRNA-based delivery approaches. While recombinant bsAbs face manufacturing complexity and short half-life, mRNA-loaded nanocarriers such as lipid nanoparticles (LNPs), poly (β-amino ester) nanoparticles (PBAE NPs), or TransIT reagents enable in situ bsAb production. These can be applied systemically or, to ex vivo cell therapy strategies where current delivery approaches rely on viral or physical transfection methods. Created in https://BioRender.com

The production of clinical-grade antibodies presents significant manufacturing challenges [80]. To overcome this limitation, in situ bsAb expression using nucleic acid-based formats is being actively investigated, using electroporation upon intramuscular injection of DNA [81], virus-based delivery approaches [82, 83] and, more recently, mRNA-loaded NPs (Fig. 5). These strategies aim to enhance therapeutic efficacy, while reducing production complexity and cost, ultimately offering scalable alternatives to conventional recombinant protein manufacturing.

### mRNA-NPs as vector for BsAb gene therapy

In 2017, Karikó, Türeci and Sahin were the first to provide compelling evidence for the feasibility of bsAb TCE-encoding mRNA. They developed three distinct TCE constructs directed against the TAAs claudin 6 and 18.2 (tight-junction proteins), and epithelial cell adhesion molecule. Following intravenous administration of mRNA encoding these bsAbs via the TransIT™ transfection reagent, expression was predominantly localized to the liver. In mice bearing subcutaneous human ovarian carcinoma xenografts, three weekly injections of 3 µg encapsulated mRNA induced complete tumor regression. In contrast, achieving comparable efficacy required ten injections of 4–7 µg of purified protein over the same period, thus validating superiority of the mRNA approach [84]. Building on this work, Stadler and Sahin developed mRNA-loaded LNPs encoding a TCE bsAb (CD3 x Claudin 6). This approach resulted in sustained liver expression and prolonged therapeutic bsAb concentrations in the bloodstream, with a half-life of 21.6 h compared to 12.2 h for the directly administered bsAb. In cynomolgus monkeys, half-lives ranged from 26.6 to 37.7 h. Weekly mRNA injections maintained therapeutic serum levels, driving regression of subcutaneous OV-90 xenografts in mice. BNT142 is now evaluated in phase I/II clinical trial for patients with claudin 6-positive advanced solid tumors (NCT05262530) [85].

Additional studies have further demonstrated that in situ production of bsAbs using mRNA NPs offers superior antitumor efficacy, extended half-life, and reduced toxicity compared to direct administration of recombinant proteins. For instance, mRNA encoding anti-EpCAM x CD3 TCE delivered via poly (β-amino ester) conjugates coated with mannose-modified poly (glutamic acid) enabled targeted transfection of immune cells, enhanced T-cell infiltration, and effective TME remodelling in a model of peritoneal carcinomatosis [86]. Similarly, LNPs encoding TCEs such as B7H3 x CD3 (tested in melanoma and other solid tumors) [87], CD19 x CD3, and GPC3 x CD3 (evaluated in hepatocellular carcinoma) [88] exhibited prolonged protein expression, improved tumor control, and reduced dosing frequency compared to recombinant proteins in both murine and non-human primate models.

In addition to intravenous injection, intratumoral administration of mRNA-LNPs has proven effective in colorectal and ovarian cancer models. Weekly low-dose injections of EpCAM x CD3 [89], or HER2 x CD3 [90] mRNA led to strong tumor growth inhibition, suggesting that local delivery can overcome systemic barriers and ensure efficacy.

Beyond classical TCEs, mRNA strategies have expanded to encode bsAbs targeting immune checkpoints or chemokines. For instance, PD-L1 x PD-1 and PD-1 x CTLA-4 bsAbs delivered via LNPs achieved prolonged expression and enhanced efficacy in intestinal and colon cancer models [91, 92]. Moreover, a liver-targeted approach using mRNA encoding a bsAb against the chemokines CCL2 and CCL5 yielded potent antitumor activity across multiple cancer models, including metastatic pancreatic and colorectal cancers [93].

Finally, co-delivery of two mRNA-encoded bsAbs (EGFR x CD3 and PD-L1 × 4-1BB) demonstrated synergistic tumor inhibition in a colorectal cancer model, highlighting the potential of multiplexed mRNA nanotherapies [94].

Although various carrier systems have been explored for RNA delivery, LNPs remain the gold-standard in gene therapy across multiple applications [95]. To date, in situ bsAb production has primarily relied on LNPs and PBAE-based NPs, as summarized in Table 3.

**Table 3:** mRNA-NPs for BsAb in vivo production

Nanocarrier	mRNA encoded bsAb type	Targets	Duration of bsAb expression (versus recombinant protein)	Tumor mouse model (route of administration)	Ref.
Polymer/lipid transfection reagent (TransIT)	TCE	CD3 x CLDN6	Pharmacologically relevant plasma levels for up to 144 h (versus < 24 h for protein)	Ovarian carcinoma xenograft (IV)	[84]
LNP	TCE	CD3 x (CLDN6)_2_	Half-life of 21.6 h (versus 12.2 h for protein)	OV-90 xenograft PBMC humanized (IV)	[85]
PBAE NP with PGA-di-mannose coating	TCE	CD3 x EpCAM	Detectable serum levels >48 h (versus < 24 h for protein)	Ovarian cancer (IV)	[86]
LNP	TCE	CD3 x B7H3	Half-life of 73 h (versus 2 h for protein)	Hematologic and solid tumor human xenografts (IV)	[96]
LNP	TCE	CD3 x CD19 and CD3 x GPC3	Detectable plasma levels for 168 h (direct comparison not included)	Hepatocellular carcinoma (IV)	[88]
LNP	TCE	CD3 x EpCAM	Not specified (direct comparison not included)	Colorectal xenograft (ITU)	[89]
LNP	TCE	CD3 x HER2	Not specified (direct comparison not included)	Ovarian cancer xenograft (ITU)	[90]
LNP	Checkpoint inhibitor	PD1 x PD-L1	Detectable plasma levels >35 days (versus 21 days for protein)	Human PD-1 and PD-L1 knock -MC38 colon adenocarcinoma (IV)	[91]
LNP	Checkpoint inhibitor	CTLA4 x PD-1	Detectable serum levels >26 days (direct comparison not included)	Colon cancer (IV)	[92]
LNP	Signaling inhibitor	CCL2 x CCL5	Detectable plasma levels for 72 h (direct comparison not included)	Liver carcinoma, liver metastasis of pancreatic and colorectal cancers (IV)	[93]
Polymer/lipid transfection reagent (TransIT)	TCE and co-stimulatory bsAb	EGFR x CD3 and PD-L1 × 4-1BB	Half-life of 18.9 h and 49.6 h (versus 0.6 h and 22.2 h for protein, respectively)	Subcutaneous colorectal carcinoma xenograft (IV)	[94]

*BsAb* bispecific antibody, *CCL* chemokine (C-C motif) ligand, *CD* cluster of differentiation, *CLDN6* claudin-6, *CTLA-4* cytotoxic T-lymphocyte–associated antigen 4, *epcam* epithelial cell adhesion molecule, *EGFR* epidermal growth factor receptor, *GPC3* glypican 3, *HER2* human epidermal growth factor receptor 2, *IV* intravenous, *ITU* intratumoral, *LNP* lipid nanoparticle, *MMP-2* matrix metalloproteinase-2, *mRNA* messenger RNA, *PBAE* poly(β-amino ester), *PBMC* peripheral blood mononuclear cell, *PD-1* programmed death 1, *PD-L1* programmed death ligand 1, *PEG* polyethylene glycol, *PGA* polyglutamic acid, *TCE* T cell engager

### Nanotechnology enabling in situ-controlled production of bsAbs. Critical considerations and future directions

Gene therapy strategies employing mRNA-loaded NPs offer key advantages, including prolonged therapeutic effects and reduced systemic toxicity. These benefits are attributed to their capacity to achieve robust outcomes with lower and less frequent dosing compared to recombinant protein administration. However, the limited number of preclinical studies and variability in experimental designs make it currently difficult to draw definitive conclusions. Notably, reported mRNA-NP dosing regimens vary from 0.1 µg once weekly to 30 µg administered three times per week. This variability may also be influenced by multiple factors, including the delivery system, mRNA construct design, expression kinetics, administration route, and biological activity of the encoded protein. A comprehensive understanding of these parameters will be critical for successful clinical translation.

To date, most studies have utilized LNPs as delivery vehicles and relied on intravenous or intratumoral administration. However, work by Weissman and others has shown that the route of administration significantly influences the pharmacokinetics and expression profile of mRNA-encoded proteins [97]. These findings underscore the need to not only systematically investigate alternative delivery platforms, but also administration routes in the context of bsAb therapies.

In addition, advanced mRNA formats such as self-amplifying mRNA and circular RNA (circRNA) remain largely unexplored in bsAb applications. These constructs have shown promising results in improving expression kinetics and durability. For instance, circRNA encoding luciferase has achieved expression half-lives exceeding 80 h, nearly double as compared to linear mRNA [98], thereby implying significant potential for improved pharmacokinetics of mRNA-encoded bsAbs.

In the currently available literature, bsAb production is generally not targeted to a specific tissue; instead, achieving high overall levels of therapeutic protein is prioritized. This implies that their primary functional tropism towards the liver is not of concern, as virtually any cell may function as production and secretion center. This may in part explain the lack of clear selection patterns in terms of nanocarrier specifics, leaving the potent and extensively studied liver-tropic LNPs promising candidates. Particles with prominent extrahepatic tropism, such as Dan Siegwart’s lung or spleen SORT LNPs [99], have so far not been explored for the delivery of bsAb-encoding mRNA but may offer opportunities for specific applications. Site-specific production and secretion could result in higher local Ab concentrations at the target site, similar to intratumorally administered NPs.

Other innovative but unexplored strategies include the incorporation of mRNA encoding bsAbs into hydrogels or scaffolds to enable localized, controlled release [100]. Microneedle-based systems, such as mesoporous silica nanoneedles delivering minicircle DNA encoding an EpCAM x CD3 bsAb, have demonstrated effective gene delivery and antitumor activity in breast cancer models [101], suggesting these platforms could be adapted to bsAb mRNA-based strategies.

Overall, while mRNA-based in situ bsAb production is still an emerging area, the encouraging results obtained so far, and the wide range of yet-to-be-explored technologies suggest that this field holds substantial promise and offers ample opportunities for innovation and clinical advancement.

## Future insights. Nanocarriers as potential vectors for BsAb adoptive cell therapies

Genetic information can also be introduced ex vivo into patient-derived cells, as demonstrated by adoptive cell therapies like CAR (chimeric antigen receptor) modified T cells. Clinically, this involves collecting patient cells, genetically modifying them (typically via viral vectors or electroporation), expanding them, and reinfusing them [102]. This strategy is now being extended to bsAbs by engineering cells to secrete bsAbs, thereby circumventing the need for continuous protein infusion (Fig. 5). Unlike direct gene transfer (Sect. 4.1), this method benefits from the intrinsic tumor-homing capacity of certain cells. Notably, certain cell types, such as T cells, offer dual functionality by acting both as bsAb producers and effector cells [2, 103].

Following a pioneering study by Alvarez-Vallina’s group in 2003, demonstrating that ex vivo engineered human cells (embryonic kidney cells, HEK293) could secrete functionally active bsAbs in vivo [104], several methods to generate a variety of bsAb-secreting cell types with promising therapeutic effects are under active preclinical investigation. Among these, T cells engineered to secrete TCE antibodies, also known as STAb (*S*ecretion of *T*-cell redirecting *Ab*s) therapy [103], is the most prominent. Preclinical STAb studies elicited significant positive responses for the treatment of hematological malignancies targeting CD19 [105–107], B-cell maturation antigen (BCMA) [108], CD123 [109], or CD1a [110]. Currently, two STAb first-in-human clinical trials in hematological cancers are actively enrolling patients. The same approach has been applied to solid tumors, including colon carcinoma targeting the CEA [111]. Comparative studies showed that STAb-T cells outperform second generation CAR-T cells by recruiting bystander, unmodified T cells, amplifying the therapeutic effects, and inducing immunologic memory [107, 108].

Beyond T cells, mesenchymal stem cells (MSCs) have shown promise as bsAb producers due to their ease of expansion, extended lifespan, and transduction capabilities [112]. However, their immunosuppressive nature may facilitate tumor growth in vivo [113]. One strategy to mitigate this involves housing genetically modified MSCs in biomaterial scaffolds to create localized bsAb “biological minipumps,” thereby limiting systemic cell dissemination. Several studies have supported the safety, utility, and efficacy of this approach for the secretion of therapeutic proteins including bsAbs [112]. Plasma cells, known for their high secretory capacity [114], and other hematopoietic cells like NK cells [115] and macrophages [116] are also being explored for bsAb production.

All the bsAb-secreting cell types described above are currently generated ex vivo using viral vectors or electroporation. However, viral vector manufacturing is a complex, time-consuming, and costly process, representing a critical bottleneck for large-scale production and raisingconcerns such as insertional mutagenesis [117]. Electroporation is a batch-based process with limited scalability, substantial cell toxicity from electric pulses and reliance on specialized equipment [118]. Non-viral methods based on NPs could address these challenges, though their application for transfection of primary cells to create “secreting bsAb factories” has yet to be fully realized. NPs can be easily adapted for industrial-scale production. This offers a more cost-effective approach by reducing manufacturing complexity and minimizing the need for specialized facilities. Importantly, mRNA-NP-based systems circumvent the safety risks associated with viral integration and can be engineered to reduce toxicity compared to electroporation. Proof-of-concept studies in CAR-based immunotherapy have demonstrated success with nanocarriers, particularly LNPs, in generating CAR-T cells ex vivo with comparable activity to electroporation and lentiviral methods. Furthermore, NPs hold the potential to bypass ex vivo cell manipulation altogether by enabling direct in vivo engineering, an approach now advancing toward clinical evaluation [119]. Such technologies could ultimately help overcome the limitations of autologous and allogeneic therapies, including prolonged manufacturing timelines, high costs, and risk of graft-versus-host disease, paving the way for off-the-shelf treatments with straightforward redosing [120, 121].

Therefore, we suggest future applications of nanotechnology-enabled approaches for the ex vivo and in vivo generation of cell-secreted bsAbs to provide safer, more scalable, and cost-effective platforms, thereby advancing these therapeutics towards more patient-friendly treatment modalities.

## Challenges and outlook for clinical translation

The integration of nanotechnology with bsAbs technology offers complementary strategies to improve cancer therapy. Collectively, while preclinical studies demonstrate encouraging efficacy, clinical translation is in an early phase, with one ongoing trial (BNT142-01, a Phase I/II study of mRNA-LNPs encoding a CD3 × CLDN6 bsAb). Recent data from ASCO 2025 show that BNT142 has demonstrated first-in-human activity in CLDN6-positive solid tumors, including ovarian cancer, with a manageable safety profile [122]. Below, several challenges that could represent major obstacles to clinical implementation are discussed, as well as high-priority research gaps that need to be addressed.

### Manufacturing, scalability, and regulatory hurdles

BsAbs exhibit greater structural complexity than conventional antibodies, which heightens the risk of chain mispairing, aggregation, and batch-to-batch variability [123]. Incorporating bsAbs into nanocarrier systems presents significant challenges, requiring precise conjugation and preservation of functional activity to ensure reproducibility. These technical demands become even more pronounced in advanced designs, such as stimuli-responsive or co-delivery platforms, raising concerns regarding scalability. Nevertheless, recent innovations including novel antibody formats, microfluidics-based manufacturing, continuous processing, and site-specific conjugation methods are progressively overcoming these barriers. For mRNA-encoded bsAbs, the inherently shorter production cycle [124] and regulatory precedents established during the development of COVID-19 vaccines provide a favorable translational landscape. Importantly, GMP-compliant, scalable, and protective nanocarrier systems must be integrated into the design process from the outset, thereby enabling promising concepts to advance efficiently into clinically applicable products.

### Toward standardized characterization of bsAb–NP systems

Heterogeneity in experimental reporting represents a critical bottleneck. Key variables including ligand density, bsAb orientation and functionality, linker stability, and protein corona interactions critically influence biological performance, yet are often underreported. Establishing reproducibility and defining critical quality attributes will be decisive to align academic development with industrial and regulatory standards.

### Managing risks in bsAb–nanomedicine translation

Clinical application of naked bsAbs has revealed risks of CRS and neurotoxicity, and it remains unclear whether nanocarrier delivery mitigates or simply modulates these toxicities. Encapsulation could minimize systemic peaks and extend half-life, but nanocarriers may introduce additional challenges including complement activation, hepatotoxicity, and long-term immunogenicity that could affect immune tolerance and response durability [125]. Locoregional or depot-based delivery of mRNA-encoded bsAbs is emerging as a promising strategy for solid tumors, where systemic administration is constrained by toxicity. Tissue-targeted LNPs and hydrogel-based systems further enhance this approach by maximizing local exposure while reducing systemic toxicities such as CRS and neurotoxicity. Together, these advances position mRNA-bsAbs as a compelling modality for solid tumors, provided that future studies integrate PK/PD biomarkers, adaptive trial designs, and long-term immune monitoring to balance efficacy with safety.

### Bridging communities for clinical translation

The translation of bsAbs into nanocarrier formats is slow, partly due to limited communication between the therapeutic antibody and nanomedicine communities. In addition to creating new bsAb architectures, repurposing clinically validated bsAbs within delivery systems remains understudied. Only a few case studies exist, such as the use of blinatumomab loaded into CaCO₃/polydopamine nanoparticles to combine T cell engagement with local immunostimulation and photothermal therapy [74]. Concurrently, advances in platforms including mRNA-encoded bsAbs currently in clinical trials (BNT142 and CLDN6 x CD3) [85, 122], and hydrogel or depot delivery of TCEs demonstrate that nanocarriers can enable sustained, tumor-localized activity while modulating systemic exposure. New concepts, such as bsAb-guided targeting of mRNA-LNPs, exemplify how antibody engineering and nanocarrier design can coevolve to improve tissue selectivity. A multidisciplinary framework uniting immuno-oncology, materials science, and regulatory science will be essential to standardizing CMC, PK/PD, and safety readouts and unlocking the full potential of bsAb-NP platforms. Reviews on antibody-functionalized nanocarriers for RNA therapeutics outline practical paths for such integration [126].

### High-priority research gaps and future outlook

1. Optimization of bsAb–NC design: Control bsAb orientation, minimize non-specific interactions, enable controlled release, and achieve high-yield bsAb–mRNA delivery. AI-driven modeling and computational prediction tools will be instrumental in guiding these optimizations while anticipating toxicity risks.
2. Standardized characterization: Establish robust analytical methods to quantify bsAb density, orientation, and stability, and define regulatory-quality attributes to ensure reproducibility and streamline clinical translation.
3. Pharmacokinetic control: Apply rational nanocarrier engineering to extend bsAb bioactivity, optimize administration routes, and explore extrahepatic targeting strategies to maximize efficacy while minimizing systemic toxicity.
4. Toxicity and stability assessment: Conduct rigorous safety evaluation, including risks of CRS or neurotoxicity, while leveraging innovative delivery approaches such as locoregional or depot systems. Equally critical is assessing long term immunogenicity and its implications for immune tolerance, which remains poorly understood.
5. Sustainability and access: Broad adoption of bsAb–nanotechnology platforms will require scalable, reproducible, and cost-effective manufacturing pipelines to ensure accessibility across healthcare systems.

Collectively, addressing these research gaps will not only accelerate the bench‑to‑bedside translation of bsAb–nanotechnology platforms but also establish a foundation for next-generation immunotherapies that are safer, longer‑lasting, and broadly accessible. Importantly, advances in antibody engineering, mRNA delivery, and nanotechnology manufacturing pipelines are already converging, offering a unique window of opportunity. By embracing multidisciplinary collaboration and embedding regulatory foresight bsAb–NP systems can transcend the current limitations. If realized, this convergence has the potential to redefine precision oncology and deliver transformative treatments for patients.

## Data Availability

Data sharing is not applicable to this article as no datasets were generated or analysed during the current study.

## References

[1] Klein C, Brinkmann U, Reichert JM, Kontermann RE. The present and future of bispecific antibodies for cancer therapy. Nat Rev Drug Discov. 2024;23:301–19. 10.1038/s41573-024-00896-6. 38448606 10.1038/s41573-024-00896-6

[2] Guo X, Wu Y, Xue Y, Xie N, Shen G. Revolutionizing cancer immunotherapy: unleashing the potential of bispecific antibodies for targeted treatment. Front Immunol. 2023;14:1291836. 10.3389/fimmu.2023.1291836. 38106416 10.3389/fimmu.2023.1291836 PMC10722299

[3] Blanco B, Domínguez-Alonso C, Alvarez-Vallina L. Bispecific immunomodulatory antibodies for cancer immunotherapy. Clin Cancer Res. 2021;27:5457–64. 10.1158/1078-0432.CCR-20-3770. 34108185 10.1158/1078-0432.CCR-20-3770 PMC9306338

[4] Beishenaliev A, Loke YL, Goh SJ, Geo HN, Mugila M, Misran M, et al. Bispecific antibodies for targeted delivery of anti-cancer therapeutic agents: a review. J Control Release. 2023;359:268–86. 10.1016/j.jconrel.2023.05.032. 37244297 10.1016/j.jconrel.2023.05.032

[5] Hong Y, Nam S-M, Moon A. Antibody-drug conjugates and bispecific antibodies targeting cancers: applications of click chemistry. Arch Pharm Res. 2023;46:131–48. 10.1007/s12272-023-01433-6. 36877356 10.1007/s12272-023-01433-6

[6] Baeuerle PA, Wesche H. T-cell-engaging antibodies for the treatment of solid tumors: challenges and opportunities. Curr Opin Oncol. 2022;34:552–8. 10.1097/CCO.0000000000000869. 35880455 10.1097/CCO.0000000000000869 PMC9415207

[7] Nathan P, Hassel JC, Rutkowski P, Baurain J-F, Butler MO, Schlaak M, et al. Overall survival benefit with tebentafusp in metastatic uveal melanoma. N Engl J Med. 2021;385:1196–206. 10.1056/NEJMoa2103485. 34551229 10.1056/NEJMoa2103485

[8] Fenis A, Demaria O, Gauthier L, Vivier E, Narni-Mancinelli E. New immune cell engagers for cancer immunotherapy. Nat Rev Immunol. 2024;24(7):471–86. 10.1038/s41577-023-00982-7. 38273127 10.1038/s41577-023-00982-7

[9] Wei J, Yang Y, Wang G, Liu M. Current landscape and future directions of bispecific antibodies in cancer immunotherapy. Front Immunol. 2022;13:1035276. 10.3389/fimmu.2022.1035276. 36389699 10.3389/fimmu.2022.1035276 PMC9650279

[10] Park K, Haura EB, Leighl NB, Mitchell P, Shu CA, Girard N, et al. Amivantamab in EGFR exon 20 insertion-mutated non-small-cell lung cancer progressing on platinum chemotherapy: initial results from the CHRYSALIS phase I study. J Clin Oncol. 2021;39:3391–402. 10.1200/JCO.21.00662. 34339292 10.1200/JCO.21.00662 PMC8791812

[11] Zhang T, Lin Y, Gao Q. Bispecific antibodies targeting immunomodulatory checkpoints for cancer therapy. Cancer Biol Med. 2023;20:181–95. 10.20892/j.issn.2095-3941.2023.0002. 36971124 10.20892/j.issn.2095-3941.2023.0002 PMC10038071

[12] Keam SJ. Cadonilimab. First approval. Drugs. 2022;82:1333–9. 10.1007/s40265-022-01761-9. 35986837 10.1007/s40265-022-01761-9

[13] Vaidya A, Moore S, Chatterjee S, Guerrero E, Kim M, Farbiak L, et al. Expanding RNAi to kidneys, lungs, and spleen via selective organ targeting (SORT) SiRNA lipid nanoparticles. Adv Mater. 2024;36:e2313791. 10.1002/adma.202313791. 38973655 10.1002/adma.202313791 PMC11823468

[14] Dilliard SA, Siegwart DJ. Passive, active and endogenous organ-targeted lipid and polymer nanoparticles for delivery of genetic drugs. Nat Rev Mater. 2023;8:282–300. 10.1038/s41578-022-00529-7. 36691401 10.1038/s41578-022-00529-7 PMC9850348

[15] Marques AC, Costa PJ, Velho S, Amaral MH. Functionalizing nanoparticles with cancer-targeting antibodies: a comparison of strategies. J Control Release. 2020;320:180–200. 10.1016/j.jconrel.2020.01.035. 31978444 10.1016/j.jconrel.2020.01.035

[16] Ho K-W, Liu Y-L, Liao T-Y, Liu E-S, Cheng T-L. Strategies for non-covalent attachment of antibodies to pegylated nanoparticles for targeted drug delivery. Int J Nanomed. 2024;19:10045–64. 10.2147/IJN.S479270. 10.2147/IJN.S479270 PMC11453133 39371476

[17] Nikkhoi SK, Rahbarizadeh F, Ranjbar S, Khaleghi S, Farasat A. Liposomal nanoparticle armed with bivalent bispecific single-domain antibodies, novel weapon in HER2 positive cancerous cell lines targeting. Mol Immunol. 2018;96:98–109. 10.1016/j.molimm.2018.01.010. 29549861 10.1016/j.molimm.2018.01.010

[18] Wu S-C, Chen Y-J, Wang H-C, Chou M-Y, Chang T-Y, Yuan S-S, et al. Bispecific antibody conjugated manganese-based magnetic engineered iron oxide for imaging of HER2/neu- and EGFR-expressing tumors. Theranostics. 2016;6:118–30. 10.7150/thno.13069. 26722378 10.7150/thno.13069 PMC4679359

[19] Kao C-H, Wang J-Y, Chuang K-H, Chuang C-H, Cheng T-C, Hsieh Y-C, et al. One-step mixing with humanized anti-mPEG bispecific antibody enhances tumor accumulation and therapeutic efficacy of mPEGylated nanoparticles. Biomaterials. 2014;35:9930–40. 10.1016/j.biomaterials.2014.08.032. 25212525 10.1016/j.biomaterials.2014.08.032

[20] Lin W-W, Cheng Y-A, Li C-C, Ho K-W, Chen H-J, Chen I-JU, et al. Enhancement of tumor tropism of mPEGylated nanoparticles by anti-mPEG bispecific antibody for ovarian cancer therapy. Sci Rep. 2021;11:7598. 10.1038/s41598-021-87271-2. 33828191 10.1038/s41598-021-87271-2 PMC8027450

[21] Cheng Y-A, Chen I-J, Su Y-C, Cheng K-W, Lu Y-C, Lin W-W, et al. Enhanced drug internalization and therapeutic efficacy of pegylated nanoparticles by one-step formulation with anti-mPEG bispecific antibody in intrinsic drug-resistant breast cancer. Biomater Sci. 2019;7:3404–17. 10.1039/c9bm00323a. 31251311 10.1039/c9bm00323a

[22] Moles E, Howard CB, Huda P, Karsa M, McCalmont H, Kimpton K, et al. Delivery of pegylated liposomal doxorubicin by bispecific antibodies improves treatment in models of high-risk childhood leukemia. Sci Transl Med. 2023;15:696. 10.1126/scitranslmed.abm1262. 10.1126/scitranslmed.abm1262 37196067

[23] Howard CB, Fletcher N, Houston ZH, Fuchs AV, Boase NRB, Simpson JD, et al. Overcoming instability of antibody-nanomaterial conjugates: next generation targeted nanomedicines using bispecific antibodies. Adv Healthc Mater. 2016;5:2055–68. 10.1002/adhm.201600263. 27283923 10.1002/adhm.201600263

[24] Cui J, Ju Y, Houston ZH, Glass JJ, Fletcher NL, Alcantara S, et al. Modulating targeting of poly(ethylene glycol) particles to tumor cells using bispecific antibodies. Adv Healthc Mater. 2019;8:e1801607. 10.1002/adhm.201801607. 30868751 10.1002/adhm.201801607

[25] Lim M, Fletcher NL, Saunus JM, McCart Reed AE, Chittoory H, Simpson PT, et al. Targeted hyperbranched nanoparticles for delivery of doxorubicin in breast cancer brain metastasis. Mol Pharm. 2023;20:6169–83. 10.1021/acs.molpharmaceut.3c00558. 37970806 10.1021/acs.molpharmaceut.3c00558 PMC10699306

[26] Janowicz PW, Houston ZH, Bunt J, Fletcher NL, Bell CA, Cowin G, et al. Understanding nanomedicine treatment in an aggressive spontaneous brain cancer model at the stage of early blood brain barrier disruption. Biomaterials. 2022;283:121416. 10.1016/j.biomaterials.2022.121416. 35217483 10.1016/j.biomaterials.2022.121416

[27] Su C-Y, Chen M, Chen L-C, Ho Y-S, Ho H-O, Lin S-Y, et al. Bispecific antibodies (anti-mPEG/anti-HER2) for active tumor targeting of docetaxel (DTX)-loaded mPEGylated nanocarriers to enhance the chemotherapeutic efficacy of HER2-overexpressing tumors. Drug Deliv. 2018;25:1066–79. 10.1080/10717544.2018.1466936. 29718725 10.1080/10717544.2018.1466936 PMC6058516

[28] Cheng W-J, Lin S-Y, Chen M, Chen L-C, Ho H-O, Chuang K-H, et al. Active tumoral/tumor environmental dual-targeting by non-covalently arming with trispecific antibodies or dual-bispecific antibodies on docetaxel-loaded mPEGylated nanocarriers to enhance chemotherapeutic efficacy and minimize systemic toxicity. Int J Nanomed. 2021;16:4017–30. 10.2147/IJN.S301237. 10.2147/IJN.S301237 PMC8203191 34140769

[29] Cheng W-J, Lin S-Y, Chuang K-H, Chen M, Ho H-O, Chen L-C, et al. Combined docetaxel/pictilisib-loaded mPEGylated nanocarriers with dual HER2 targeting antibodies for synergistic chemotherapy of breast cancer. Int J Nanomed. 2022;17:5353–74. 10.2147/IJN.S388066. 10.2147/IJN.S388066 PMC9677924 36419719

[30] Zhao Y, Fletcher NL, Gemmell A, Houston ZH, Howard CB, Blakey I, et al. Investigation of the therapeutic potential of a synergistic delivery system through dual controlled release of camptothecin–doxorubicin. Adv Ther. 2020;3:1900202. 10.1002/adtp.201900202.

[31] Zhang Y, Gu X, Huang L, Yang Y, He J. Enhancing precision medicine: bispecific antibody-mediated targeted delivery of lipid nanoparticles for potential cancer therapy. Int J Pharm. 2024;654:123990. 10.1016/j.ijpharm.2024.123990. 38467208 10.1016/j.ijpharm.2024.123990

[32] Logan A, Howard CB, Huda P, Kimpton K, Ma Z, Thurecht KJ, et al. Targeted delivery of polo-like kinase 1 siRNA nanoparticles using an EGFR-PEG bispecific antibody inhibits proliferation of high-risk neuroblastoma. J Control Release. 2024;367:806–20. 10.1016/j.jconrel.2024.02.007. 38341177 10.1016/j.jconrel.2024.02.007

[33] Amabile A, Phelan M, Yu Z, Silva P, Marks A, Morla-Folch J, et al. Bispecific antibody targeting of lipid nanoparticles. Preprint [bioRxiv]. 2024. 10.1101/2024.12.20.629467.

[34] Su Y-C, Burnouf P-A, Chuang K-H, Chen B-M, Cheng T-L, Roffler SR. Conditional internalization of pegylated nanomedicines by PEG engagers for triple negative breast cancer therapy. Nat Commun. 2017;8:15507. 10.1038/ncomms15507. 28593948 10.1038/ncomms15507 PMC5472176

[35] Chen I-J, Cheng Y-A, Ho K-W, Lin W-W, Cheng K-W, Lu Y-C, et al. Bispecific antibody (HER2 × mPEG) enhances anti-cancer effects by precise targeting and accumulation of mPEGylated liposomes. Acta Biomater. 2020;111:386–97. 10.1016/j.actbio.2020.04.029. 32417267 10.1016/j.actbio.2020.04.029

[36] Parker CL, McSweeney MD, Lucas AT, Jacobs TM, Wadsworth D, Zamboni WC, et al. Pretargeted delivery of PEG-coated drug carriers to breast tumors using multivalent, bispecific antibody against polyethylene glycol and HER2. Nanomedicine. 2019;21:102076. 10.1016/j.nano.2019.102076. 31394261 10.1016/j.nano.2019.102076 PMC7224238

[37] Ho K-W, Chen I-JU, Cheng Y-A, Liao T-Y, Liu E-S, Chen H-J, et al. Double attack strategy for leukemia using a pre-targeting bispecific antibody (CD20 Ab-mPEG scFv) and actively attracting pegylated liposomal doxorubicin to enhance anti-tumor activity. J Nanobiotechnol. 2021;19:16. 10.1186/s12951-020-00752-w. 10.1186/s12951-020-00752-w PMC7796588 33422061

[38] Dietmair B, Humphries J, Mercer TR, Thurecht KJ, Howard CB, Cheetham SW. Targeted mRNA delivery with bispecific antibodies that tether LNPs to cell-surface markers. Preprint [bioRxiv]. 2024. 10.1101/2024.10.17.618962. 10.1016/j.omtn.2025.102520 PMC11999258 40235853

[39] Sivaram AJ, Wardiana A, Howard CB, Mahler SM, Thurecht KJ. Recent advances in the generation of antibody-nanomaterial conjugates. Adv Healthc Mater. 2018;7:1700607. 10.1002/adhm.201700607. 10.1002/adhm.201700607 28961378

[40] Van Lith SAM, Van Duijnhoven SMJ, Navis AC, Leenders WPJ, Dolk E, Wennink JWH, et al. Legomedicine- a versatile chemo-enzymatic approach for the preparation of targeted dual-labeled Llama antibody–nanoparticle conjugates. Bioconjug Chem. 2017;28:539–48. 10.1021/acs.bioconjchem.6b00638. 28045502 10.1021/acs.bioconjchem.6b00638 PMC5330650

[41] Cheng Y-A, Wu T-H, Wang Y-M, Cheng T-L, Chen I-J, Lu Y-C, et al. Humanized bispecific antibody (mPEG × HER2) rapidly confers pegylated nanoparticles tumor specificity for multimodality imaging in breast cancer. J Nanobiotechnol. 2020;18:118. 10.1186/s12951-020-00680-9. 10.1186/s12951-020-00680-9 PMC7457265 32854720

[42] O’Brien MER, Wigler N, Inbar M, Rosso R, Grischke E, Santoro A, et al. Reduced cardiotoxicity and comparable efficacy in a phase III trial of pegylated liposomal doxorubicin HCl (CAELYX/Doxil) versus conventional doxorubicin for first-line treatment of metastatic breast cancer. Ann Oncol. 2004;15:440–9. 10.1093/annonc/mdh097. 14998846 10.1093/annonc/mdh097

[43] Barenholz Y. Doxil^®^-the first FDA-approved nano-drug: lessons learned. J Control Release. 2012;160:117–34. 10.1016/j.jconrel.2012.03.020. 22484195 10.1016/j.jconrel.2012.03.020

[44] d’Avanzo N, Celia C, Barone A, Carafa M, Di Marzio L, Santos HA, et al. Immunogenicity of polyethylene glycol based nanomedicines: mechanisms, clinical implications and systematic approach. Adv Ther. 2020;3:1900170. 10.1002/adtp.201900170.

[45] López-Estévez AM, Lapuhs P, Pineiro‐Alonso L, Alonso MJ. Personalized cancer nanomedicine: overcoming biological barriers for intracellular delivery of biopharmaceuticals. Adv Mater. 2024;36:2309355. 10.1002/adma.202309355. 10.1002/adma.202309355 38104275

[46] Zhang Z, Sun H, Giannino J, Wu Y, Cheng C. Biodegradable zwitterionic polymers as PEG alternatives for drug delivery. J Polym Sci. 2024;62(10):2231–50. 10.1002/pol.20230916. 10.1002/pol.20230916 PMC11376432 39247254

[47] Nogueira SS, Schlegel A, Maxeiner K, Weber B, Barz M, Schroer MA, et al. Polysarcosine-functionalized lipid nanoparticles for therapeutic mRNA delivery. ACS Appl Nano Mater. 2020;3:10634–45. 10.1021/acsanm.0c01834.

[48] Liu L, Pan D, Chen S, Martikainen M-V, Kårlund A, Ke J, et al. Systematic design of cell membrane coating to improve tumor targeting of nanoparticles. Nat Commun. 2022;13:6181. 10.1038/s41467-022-33889-3. 36261418 10.1038/s41467-022-33889-3 PMC9580449

[49] Macário-Soares A, Sousa‐Oliveira I, Correia M, Pires PC, Sharma A, Kumar Jha N, et al. Cell membrane and extracellular vesicle membrane‐coated nanoparticles: an envisaged approach for the management of skin conditions. View. 2024;5:20240043. 10.1002/VIW.20240043.

[50] Xu F, Jiang D, Xu J, Dai H, Fan Q, Fei Z, et al. Engineering of dendritic cell bispecific extracellular vesicles for tumor-targeting immunotherapy. Cell Rep. 2023;42:113138. 10.1016/j.celrep.2023.113138. 37738123 10.1016/j.celrep.2023.113138

[51] Jallinoja VIJ, Houghton JL. Current landscape in clinical pretargeted radioimmunoimaging and therapy. J Nucl Med. 2021;62:1200–6. 10.2967/jnumed.120.260687. 34016727 10.2967/jnumed.120.260687 PMC8882889

[52] Sharkey RM, Karacay H, Richel H, McBride WJ, Rossi EA, Chang K, et al. Optimizing bispecific antibody pretargeting for use in radioimmunotherapy. Clin Cancer Res. 2003;9:S3897–913. 14506188

[53] Han X, Zhou Y, Liu W. Precision cardio-oncology: understanding the cardiotoxicity of cancer therapy. Npj Precision Onc. 2017;1:31. 10.1038/s41698-017-0034-x. 10.1038/s41698-017-0034-x PMC5871905 29872712

[54] Géraud A, Hueso T, Laparra A, Bige N, Ouali K, Cauquil C, et al. Reactions and adverse events induced by T-cell engagers as anti-cancer immunotherapies, a comprehensive review. Eur J Cancer. 2024;205:114075. 10.1016/j.ejca.2024.114075. 38733717 10.1016/j.ejca.2024.114075

[55] Haber L, Olson K, Kelly MP, Crawford A, DiLillo DJ, Tavaré R, et al. Generation of T-cell-redirecting bispecific antibodies with differentiated profiles of cytokine release and biodistribution by CD3 affinity tuning. Sci Rep. 2021;11(1):14397. 10.1038/s41598-021-93842-0. 34257348 10.1038/s41598-021-93842-0 PMC8277787

[56] Dang K, Castello G, Clarke SC, Li Y, Balasubramani A, Boudreau A, et al. Attenuating CD3 affinity in a PSMAxCD3 bispecific antibody enables killing of prostate tumor cells with reduced cytokine release. J Immunother Cancer. 2021;9:e002488. 10.1136/jitc-2021-002488. 34088740 10.1136/jitc-2021-002488 PMC8183203

[57] Middelburg J, Kemper K, Engelberts P, Labrijn AF, Schuurman J, van Hall T. Overcoming challenges for CD3-bispecific antibody therapy in solid tumors. Cancers (Basel). 2021;13:287. 10.3390/cancers13020287. 33466732 10.3390/cancers13020287 PMC7829968

[58] Wu L, Seung E, Xu L, Rao E, Lord DM, Wei RR, et al. Trispecific antibodies enhance the therapeutic efficacy of tumor-directed T cells through T cell receptor co-stimulation. Nat Cancer. 2020;1:86–98. 10.1038/s43018-019-0004-z. 35121834 10.1038/s43018-019-0004-z

[59] Ai Z, Wang B, Song Y, Cheng P, Liu X, Sun P. Prodrug-based bispecific antibodies for cancer therapy: advances and future directions. Front Immunol. 2025;16:1523693. 10.3389/fimmu.2025.1523693. 39911391 10.3389/fimmu.2025.1523693 PMC11794264

[60] McCue AC, Demarest SJ, Froning KJ, Hickey MJ, Antonysamy S, Kuhlman B. Engineering a tumor-selective prodrug T-cell engager bispecific antibody for safer immunotherapy. MAbs. 2024;16:2373325. 10.1080/19420862.2024.2373325. 38962811 10.1080/19420862.2024.2373325 PMC11225918

[61] Boustany LM, LaPorte SL, Wong L, White C, Vinod V, Shen J, et al. A probody T cell-engaging bispecific antibody targeting EGFR and CD3 inhibits colon cancer growth with limited toxicity. Cancer Res. 2022;82:4288–98. 10.1158/0008-5472.CAN-21-2483. 36112781 10.1158/0008-5472.CAN-21-2483 PMC9664135

[62] Gong N, Han X, Xue L, Billingsley MM, Huang X, El-Mayta R, et al. Small-molecule-mediated control of the anti-tumour activity and off-tumour toxicity of a supramolecular bispecific T cell engager. Nat Biomed Eng. 2024;8:513–28. 10.1038/s41551-023-01147-6. 38378820 10.1038/s41551-023-01147-6

[63] Zhu M, Wu B, Brandl C, Johnson J, Wolf A, Chow A, et al. Blinatumomab, a bispecific t-cell engager (BiTE^®^) for CD-19 targeted cancer immunotherapy: clinical pharmacology and its implications. Clin Pharmacokinet. 2016;55:1271–88. 10.1007/s40262-016-0405-4. 27209293 10.1007/s40262-016-0405-4

[64] Zorzi A, Linciano S, Angelini A. Non-covalent albumin-binding ligands for extending the circulating half-life of small biotherapeutics. Medchemcomm. 2019;10:1068–81. 10.1039/c9md00018f. 31391879 10.1039/c9md00018f PMC6644573

[65] Cheng Q, Zhang X-N, Li J, Chen J, Wang Y, Zhang Y. Synthesis of bispecific antibody conjugates using functionalized poly-ADP-ribose polymers. Biochemistry. 2023;62:1138–44. 10.1021/acs.biochem.2c00718. 36821831 10.1021/acs.biochem.2c00718 PMC10033384

[66] Zhang W, Li D, Xu X, Chen Y, Shi X, Pan Y, et al. A bispecific peptide-polymer conjugate bridging target-effector cells to enhance immunotherapy. Adv Healthc Mater. 2023;12:e2202977. 10.1002/adhm.202202977. 36878223 10.1002/adhm.202202977

[67] Leconet W, Liu H, Guo M, Le Lamer-Déchamps S, Molinier C, Kim S, et al. Anti-PSMA/CD3 bispecific antibody delivery and antitumor activity using a polymeric depot formulation. Mol Cancer Ther. 2018;17:1927–40. 10.1158/1535-7163.MCT-17-1138. 29891487 10.1158/1535-7163.MCT-17-1138

[68] Wei P-S, Chen Y-J, Lin S-Y, Chuang K-H, Sheu M-T, Ho H-O. In situ subcutaneously injectable thermosensitive PEG-PLGA diblock and PLGA-PEG-PLGA triblock copolymer composite as sustained delivery of bispecific anti-CD3 ScFv T-cell/anti-EGFR fab engager (BiTEE). Biomaterials. 2021;278:121166. 10.1016/j.biomaterials.2021.121166. 34634663 10.1016/j.biomaterials.2021.121166

[69] Alhallak K, Sun J, Wasden K, Guenthner N, O’Neal J, Muz B, et al. Nanoparticle T-cell engagers as a modular platform for cancer immunotherapy. Leukemia. 2021;35:2346–57. 10.1038/s41375-021-01127-2. 33479469 10.1038/s41375-021-01127-2 PMC8292428

[70] Xu M, Wen Y, Liu Y, Tan X, Chen X, Zhu X, et al. Hollow mesoporous ruthenium nanoparticles conjugated bispecific antibody for targeted anti-colorectal cancer response of combination therapy. Nanoscale. 2019;11:9661–78. 10.1039/C9NR01904A. 31065660 10.1039/c9nr01904a

[71] Cheng W-J, Chuang K-H, Lo Y-J, Chen M, Chen Y-J, Roffler SR, et al. Bispecific T-cell engagers non-covalently decorated drug-loaded pegylated nanocarriers for cancer immunochemotherapy. J Control Release. 2022;344:235–48. 10.1016/j.jconrel.2022.03.015. 35288168 10.1016/j.jconrel.2022.03.015

[72] Linderman SW, DeRidder L, Sanjurjo L, Foote MB, Alonso MJ, Kirtane AR, et al. Enhancing immunotherapy with tumour-responsive nanomaterials. Nat Rev Clin Oncol. 2025;22:262–82. 10.1038/s41571-025-01000-6. 40050505 10.1038/s41571-025-01000-6

[73] Fan R, Chen C, Mu M, Chuan D, Liu H, Hou H, et al. Engineering MMP-2 activated nanoparticles carrying B7-H3 bispecific antibodies for ferroptosis-enhanced glioblastoma immunotherapy. ACS Nano. 2023;17:9126–39. 10.1021/acsnano.2c12217. 37097811 10.1021/acsnano.2c12217

[74] Li R, Shi X, Zhang J, Liu B, Shen J, Liu H, et al. CaCO3-encapsulated polydopamine with an adsorbed TLR7 agonist for improved tumor photothermal immunotherapy. Heliyon. 2024;10:e33837. 10.1016/j.heliyon.2024.e33837. 39050425 10.1016/j.heliyon.2024.e33837 PMC11268191

[75] Xu G, Luo Y, Wang H, Wang Y, Liu B, Wei J. Therapeutic bispecific antibodies against intracellular tumor antigens. Cancer Lett. 2022;538:215699. 10.1016/j.canlet.2022.215699. 35487312 10.1016/j.canlet.2022.215699

[76] Middleton MR, McAlpine C, Woodcock VK, Corrie P, Infante JR, Steven NM, et al. Tebentafusp, a TCR/anti-CD3 bispecific fusion protein targeting gp100, potently activated antitumor immune responses in patients with metastatic melanoma. Clin Cancer Res. 2020;26:5869–78. 10.1158/1078-0432.CCR-20-1247. 32816891 10.1158/1078-0432.CCR-20-1247 PMC9210997

[77] Douglass J, Hsiue EH-C, Mog BJ, Hwang MS, DiNapoli SR, Pearlman AH, et al. Bispecific antibodies targeting mutant RAS neoantigens. Sci Immunol. 2021;6:57. 10.1126/sciimmunol.abd5515. 10.1126/sciimmunol.abd5515 PMC8141259 33649101

[78] Ebrahimi S, Lohnes BJ, Khan SA, Peipp M, Bockamp E, Klein C, et al. Targeting mutated KRAS by HLA-A*02:01 restricted anti-KRAS TCR-mimic CAR and bispecific T cell engager. J Mol Med. 2025;103:1231–47. 10.1007/s00109-025-02585-2. 40794198 10.1007/s00109-025-02585-2 PMC12449438

[79] López-Estévez AM, Sanjurjo L, Turrero Á, Arriaga I, Abrescia NGA, Poveda A, et al. Nanotechnology-assisted intracellular delivery of antibody as a precision therapy approach for KRAS-driven tumors. J Control Release. 2024;373:277–92. 10.1016/j.jconrel.2024.07.032. 39019086 10.1016/j.jconrel.2024.07.032

[80] Salih HR, Jung G. The challenges of translation. EMBO Mol Med. 2019;11:e10874. 10.15252/emmm.201910874. 31625285 10.15252/emmm.201910874 PMC6895598

[81] Bhojnagarwala PS, O’Connell RP, Park D, Liaw K, Ali AR, Bordoloi D, et al. In vivo DNA-launched bispecific T cell engager targeting IL-13Rα2 controls tumor growth in an animal model of glioblastoma multiforme. Mol Ther. 2022;26:289–301. 10.1016/j.omto.2022.07.003. 10.1016/j.omto.2022.07.003 PMC9418050 36090479

[82] De Sostoa J, Fajardo CA, Moreno R, Ramos MD, Farrera-Sal M, Alemany R. Targeting the tumor stroma with an oncolytic adenovirus secreting a fibroblast activation protein-targeted bispecific T-cell engager. J Immunother Cancer. 2019;7:1–15. 10.1186/s40425-019-0505-4. 30683154 10.1186/s40425-019-0505-4 PMC6347837

[83] Basnet S, Santos JM, Quixabeira DCA, Clubb JHA, Grönberg-Vähä-Koskela SAM, Arias V, et al. Oncolytic adenovirus coding for bispecific T cell engager against human MUC-1 potentiates T cell response against solid tumors. Mol Ther Oncolytics. 2023;28:59–73. 10.1016/j.omto.2022.12.007. 36699617 10.1016/j.omto.2022.12.007 PMC9842968

[84] Stadler CR, Bähr-Mahmud H, Celik L, Hebich B, Roth AS, Roth RP, et al. Elimination of large tumors in mice by mRNA-encoded bispecific antibodies. Nat Med. 2017;23:815–7. 10.1038/nm.4356. 28604701 10.1038/nm.4356

[85] Stadler CR, Ellinghaus U, Fischer L, Bähr-Mahmud H, Rao M, Lindemann C, et al. Preclinical efficacy and pharmacokinetics of an RNA-encoded T cell-engaging bispecific antibody targeting human Claudin 6. Sci Transl Med. 2024;16:1–13. 10.1126/scitranslmed.adl2720. 10.1126/scitranslmed.adl2720 38776391

[86] Hao S, Inamdar VV, Sigmund EC, Zhang F, Stephan SB, Watson C, et al. BiTE secretion from in situ-programmed myeloid cells results in tumor-retained pharmacology. J Control Release. 2022;342:14–25. 10.1016/j.jconrel.2021.12.029. 34953983 10.1016/j.jconrel.2021.12.029 PMC8840964

[87] Chapoval AI, Ni J, Lau JS, Wilcox RA, Flies DB, Liu D, et al. B7-H3: a costimulatory molecule for T cell activation and IFN-γ production. Nat Immunol. 2001;2:269–74. 10.1038/85339. 11224528 10.1038/85339

[88] Kai X, Zhang Y, Wei B, Tatang D, Angus S, Jin C et al. Tissue-targeted lipid nanoparticle delivery for mRNA encoding bispecific T-cell engager demonstrated potent antitumor effects on both hematological malignancies and solid tumors. J Immunother Cancer. 202;11:A1512–3. 10.1136/jitc-2023-SITC2023.1358. Meeting Abstract: SITC 38th Annual Meeting .

[89] Golubovskaya V, Sienkiewicz J, Sun J, Huang Y, Hu L, Zhou H, et al. mRNA-lipid nanoparticle (LNP) delivery of humanized EpCAM-CD3 bispecific antibody significantly blocks colorectal cancer tumor growth. Cancers. 2023;15:2860. 10.3390/cancers15102860. 37345198 10.3390/cancers15102860 PMC10216523

[90] Hu L, Zhang S, Sienkiewicz J, Zhou H, Berahovich R, Sun J, et al. HER2-CD3-Fc bispecific antibody-encoding mRNA delivered by lipid nanoparticles suppresses HER2-positive tumor growth. Vaccines. 2024;12:808. 10.3390/vaccines12070808. 39066446 10.3390/vaccines12070808 PMC11281407

[91] Wu L, Wang W, Tian J, Qi C, Cai Z, Yan W, et al. Engineered mRNA-expressed bispecific antibody prevent intestinal cancer via lipid nanoparticle delivery. Bioengineered. 2021;12:12383–93. 10.1080/21655979.2021.2003666. 34895063 10.1080/21655979.2021.2003666 PMC8810065

[92] Zeng J, Fang Y, Zhang Z, Lv Z, Wang X, Huang Q, et al. Antitumor activity of Z15-0-2, a bispecific nanobody targeting PD-1 and CTLA-4. Oncogene. 2024;43:2244–52. 10.1038/s41388-024-03066-5. 38806619 10.1038/s41388-024-03066-5 PMC11245388

[93] Wang Y, Tiruthani K, Li S, Hu M, Zhong G, Tang Y, et al. mRNA delivery of a bispecific single-domain antibody to polarize tumor-associated macrophages and synergize immunotherapy against liver malignancies. Adv Mater. 2021;33:1–11. 10.1002/adma.202007603. 10.1002/adma.202007603 PMC8240965 33945178

[94] Hangiu O, Navarro R, Frago S, Rubio-Pérez L, Tapia-Galisteo A, Díez-Alonso L, et al. Effective cancer immunotherapy combining mRNA-encoded bispecific antibodies that induce polyclonal T cell engagement and PD-L1-dependent 4-1BB costimulation. Front Immunol. 2025;15:1494206. 10.3389/fimmu.2024.1494206. 39835115 10.3389/fimmu.2024.1494206 PMC11743637

[95] Hou X, Zaks T, Langer R, Dong Y. Lipid nanoparticles for mRNA delivery. Nat Rev Mater. 2021;6:1078–94. 10.1038/s41578-021-00358-0. 34394960 10.1038/s41578-021-00358-0 PMC8353930

[96] Huang C, Duan X, Wang J, Tian Q, Ren Y, Chen K, et al. Lipid nanoparticle delivery system for Mrna encoding B7H3-redirected bispecific antibody displays potent antitumor effects on malignant tumors. Adv Sci. 2023;10:1–16. 10.1002/advs.202205532. 10.1002/advs.202205532 PMC9875623 36403209

[97] Pardi N, Tuyishime S, Muramatsu H, Kariko K, Mui BL, Tam YK, et al. Expression kinetics of nucleoside-modified mRNA delivered in lipid nanoparticles to mice by various routes. J Control Release. 2015;217:345–51. 10.1016/j.jconrel.2015.08.007. 26264835 10.1016/j.jconrel.2015.08.007 PMC4624045

[98] Wesselhoeft RA, Kowalski PS, Anderson DG. Engineering circular RNA for potent and stable translation in eukaryotic cells. Nat Commun. 2018;9:2629. 10.1038/s41467-018-05096-6. 29980667 10.1038/s41467-018-05096-6 PMC6035260

[99] Cheng Q, Wei T, Farbiak L, Johnson LT, Dilliard SA, Siegwart DJ. Selective organ targeting (SORT) nanoparticles for tissue-specific mRNA delivery and CRISPR–Cas gene editing. Nat Nanotechnol. 2020;15:313–20. 10.1038/s41565-020-0669-6. 32251383 10.1038/s41565-020-0669-6 PMC7735425

[100] Zhong R, Talebian S, Mendes BB, Wallace G, Langer R, Conde J, et al. Hydrogels for RNA delivery. Nat Mater. 2023;22:818–31. 10.1038/s41563-023-01472-w. 36941391 10.1038/s41563-023-01472-w PMC10330049

[101] Cai J, Jiang S, Liao J, Fan H, Peng C, Shi S, et al. Manganese-doped biostimulatory nanoneedle for MRI-visual bispecific antibody gene delivery and immunosuppression reversal as a cancer immunotherapy strategy. Chem Eng J. 2023;462:142242. 10.1016/j.cej.2023.142242.

[102] Ayala Ceja M, Khericha M, Harris CM, Puig-Saus C, Chen YY. CAR-T cell manufacturing: major process parameters and next-generation strategies. J Exp Med. 2024;221:e20230903. 10.1084/jem.20230903. 38226974 10.1084/jem.20230903 PMC10791545

[103] Blanco B, Compte M, Lykkemark S, Sanz L, Alvarez-Vallina L. T cell-redirecting strategies to stab tumors: beyond cars and bispecific antibodies. Trends Immunol. 2019;40:243–57. 10.1016/j.it.2019.01.008. 30827461 10.1016/j.it.2019.01.008

[104] Blanco B, Holliger P, Vile RG, Alvarez-Vallina L. Induction of human T lymphocyte cytotoxicity and Inhibition of tumor growth by tumor-specific diabody-based molecules secreted from gene-modified bystander cells. J Immunol. 2003;171:1070–7. 10.4049/jimmunol.171.2.1070. 12847281 10.4049/jimmunol.171.2.1070

[105] Blanco B, Ramírez-Fernández Á, Bueno C, Argemí-Muntadas L, Fuentes P, Aguilar-Sopeña Ó, et al. Overcoming CAR-mediated CD19 downmodulation and leukemia relapse with T lymphocytes secreting anti-CD19 T-cell engagers. Cancer Immunol Res. 2022;10:498–511. 10.1158/2326-6066.CIR-21-0853. 35362043 10.1158/2326-6066.CIR-21-0853 PMC7612571

[106] Liu X, Barrett DM, Jiang S, Fang C, Kalos M, Grupp SA, et al. Improved anti-leukemia activities of adoptively transferred T cells expressing bispecific T-cell engager in mice. Blood Cancer J. 2016;6:e430. 10.1038/bcj.2016.38. 27258611 10.1038/bcj.2016.38 PMC5141353

[107] Velasquez MP, Torres D, Iwahori K, Kakarla S, Arber C, Rodriguez-Cruz T, et al. T cells expressing CD19-specific engager molecules for the immunotherapy of CD19-positive malignancies. Sci Rep. 2016;6:27130. 10.1038/srep27130. 27255991 10.1038/srep27130 PMC4891739

[108] Díez-Alonso L, Falgas A, Arroyo-Ródenas J, Romencín PA, Martínez A, Gómez-Rosel M, et al. Engineered T cells secreting anti-BCMA T cell engagers control multiple myeloma and promote immune memory in vivo. Sci Transl Med. 2024;16:734. 10.1126/scitranslmed.adg7962. 10.1126/scitranslmed.adg7962 38354229

[109] Bonifant CL, Szoor A, Torres D, Joseph N, Velasquez MP, Iwahori K, et al. CD123-engager T cells as a novel immunotherapeutic for acute myeloid leukemia. Mol Ther. 2016;24:1615–26. 10.1038/mt.2016.116. 27401038 10.1038/mt.2016.116 PMC5113097

[110] Jiménez-Reinoso A, Tirado N, Martinez-Moreno A, Díaz VM, García-Peydró M, Hangiu O, et al. Efficient preclinical treatment of cortical T cell acute lymphoblastic leukemia with T lymphocytes secreting anti-CD1a T cell engagers. J Immunother Cancer. 2022;10:e005333. 10.1136/jitc-2022-005333. 36564128 10.1136/jitc-2022-005333 PMC9791403

[111] Compte M, Blanco B, Serrano F, Cuesta AM, Sanz L, Bernad A, et al. Inhibition of tumor growth in vivo by in situ secretion of bispecific anti-CEA x anti-CD3 diabodies from lentivirally transduced human lymphocytes. Cancer Gene Ther. 2007;14:380–8. 10.1038/sj.cgt.7701021. 17218946 10.1038/sj.cgt.7701021

[112] Compte M, Cuesta AM, Sánchez-Martín D, Alonso-Camino V, Vicario JL, Sanz L, et al. Tumor immunotherapy using gene-modified human mesenchymal stem cells loaded into synthetic extracellular matrix scaffolds. Stem Cells. 2009;27:753–60. 10.1634/stemcells.2008-0831. 19096041 10.1634/stemcells.2008-0831 PMC2729675

[113] Djouad F, Plence P, Bony C, Tropel P, Apparailly F, Sany J, et al. Immunosuppressive effect of mesenchymal stem cells favors tumor growth in allogeneic animals. Blood. 2003;102:3837–44. 10.1182/blood-2003-04-1193. 12881305 10.1182/blood-2003-04-1193

[114] Hill TF, Narvekar P, Asher G, Camp N, Thomas KR, Tasian SK, et al. Human plasma cells engineered to secrete bispecifics drive effective in vivo leukemia killing. Preprint [bioRxiv]. 2023. 10.1101/2023.08.24.554523.

[115] Zhang M, Lam K-P, Xu S. Natural killer cell engagers (NKCEs): a new frontier in cancer immunotherapy. Front Immunol. 2023;14:1207276. 10.3389/fimmu.2023.1207276. 37638058 10.3389/fimmu.2023.1207276 PMC10450036

[116] Gardell JL, Matsumoto LR, Chinn H, DeGolier KR, Kreuser SA, Prieskorn B, et al. Human macrophages engineered to secrete a bispecific T cell engager support antigen-dependent T cell responses to glioblastoma. J Immunother Cancer. 2020;8:e001202. 10.1136/jitc-2020-001202. 33122397 10.1136/jitc-2020-001202 PMC7597484

[117] Lee NK, Chang JW. Manufacturing cell and gene therapies: challenges in clinical translation. Ann Lab Med. 2024;44:314–23. 10.3343/alm.2023.0382. 38361427 10.3343/alm.2023.0382 PMC10961620

[118] Mir LM. Electroporation-based gene therapy: recent evolution in the mechanism description and technology developments. Methods Mol Biol. 2014;1121:3–23. 10.1007/978-1-4614-9632-8_1. 24510808 10.1007/978-1-4614-9632-8_1

[119] Mullard A. In vivo CAR T cells move into clinical trials. Nat Rev Drug Discov. 2024. 10.1038/d41573-024-00150-z. 39284972 10.1038/d41573-024-00150-z

[120] Depil S, Duchateau P, Grupp SA, Mufti G, Poirot L. Off-the-shelf allogeneic CAR T cells: development and challenges. Nat Rev Drug Discov. 2020;19:185–99. 10.1038/s41573-019-0051-2. 31900462 10.1038/s41573-019-0051-2

[121] Hunter TL, Bao Y, Zhang Y, Matsuda D, Riener R, Wang A, et al. In vivo CAR T cell generation to treat cancer and autoimmune disease. Science. 2025;388:1311–7. 10.1126/science.ads8473. 40536974 10.1126/science.ads8473

[122] Yap TA, Hernando-Calvo AH, Calvo E, Moreno V, Marquez R, Papadopoulos P KP, et al. First-in-human phase I/II trial evaluating BNT142, a first-in-class mRNA encoded, bispecific antibody targeting Claudin 6 (CLDN6) and CD3, in patients (pts) with CLDN6-positive advanced solid tumors. J Clin Oncol. 2025;43(16suppl):2501–2501. 10.1200/JCO.2025.43.16_suppl.2501. Meeting Abstract: 2025 ASCO Annual Meeting I.

[123] Ingavat N, Dzulkiflie N, Liew JM, Wang X, Leong E, Loh HP, et al. Investigation on environmental factors contributing to bispecific antibody stability and the reversal of self-associated aggregates. Bioresour Bioprocess. 2024;11:82. 10.1186/s40643-024-00796-y. 39177850 10.1186/s40643-024-00796-y PMC11343937

[124] Shi Y, Shi M, Wang Y, You J. Progress and prospects of mRNA-based drugs in pre-clinical and clinical applications. Sig Transduct Target Ther. 2024;9:322. 10.1038/s41392-024-02002-z. 10.1038/s41392-024-02002-z PMC11564800 39543114

[125] Fortune A, Aime A, Raymond D, Kumar S. Nanotechnology in medicine: a double-edged sword for health outcomes. Health Nanotechnol. 2025;1:9. 10.1186/s44301-025-00008-2.

[126] Nabih NW, Hassan HAFM, Preis E, Schaefer J, Babker A, Abbas AM, et al. Antibody-functionalized lipid nanocarriers for RNA-based cancer gene therapy: advances and challenges in targeted delivery. Nanoscale Adv. 2025. 10.1039/d5na00323g. 40880603 10.1039/d5na00323g PMC12371570

